# Convolutional neural network for classification of eight types of arrhythmia using 2D time–frequency feature map from standard 12-lead electrocardiogram

**DOI:** 10.1038/s41598-021-99975-6

**Published:** 2021-10-14

**Authors:** Da Un Jeong, Ki Moo Lim

**Affiliations:** 1 Kumoh National Institute of Technology, IT Convergence Engineering, Gumi, 39253 Republic of Korea; 2 Kumoh National Institute of Technology, Medical IT Convergence Engineering, Gumi, 39253 Republic of Korea

**Keywords:** Biomedical engineering, Classification and taxonomy

## Abstract

Electrocardiograms (ECGs) are widely used for diagnosing cardiac arrhythmia based on the deformation of signal shapes due to changes in various heart diseases. However, these abnormal signs may not be observed in some 12 ECG channels, depending on the location, the heart shape, and the type of cardiac arrhythmia. Therefore, it is necessary to closely and comprehensively observe ECG records acquired from 12 channel electrodes to diagnose cardiac arrhythmias accurately. In this study, we proposed a clustering algorithm that can classify persistent cardiac arrhythmia as well as episodic cardiac arrhythmias using the standard 12-lead ECG records and the 2D CNN model using the time–frequency feature maps to classify the eight types of arrhythmias and normal sinus rhythm. The standard 12-lead ECG records were provided by China Physiological Signal Challenge 2018 and consisted of 6877 patients. The proposed algorithm showed high performance in classifying persistent cardiac arrhythmias; however, its accuracy was somewhat low in classifying episodic arrhythmias. If our proposed model is trained and verified using more clinical data, we believe it can be used as an auxiliary device for diagnosing cardiac arrhythmias.

## Introduction

Electrocardiograms (ECGs) are widely used for diagnosing cardiac arrhythmia based on the deformation of signal shapes due to changes in the electrical and mechanical activity of the heart as a result of various heart diseases^1,2^. The standard 12-lead ECG records the electrical activity of the heart in four directions: inferior (lead II, III, and aVF), anterior (lead I, aVL, V1, and V2), septal (V3 and V4), and lateral (V5 and V6). Therefore, the signal's shape in each channel varies according to the type of cardiac arrhythmia, and abnormal symptoms may be observed only in a specific channel among the 12 channels. Among these cardiac arrhythmias, abnormal symptoms are continuously observed on the ECG as persistent symptoms. Some signs are also observed intermittently with normal signals^2^. Therefore, it is not easy to detect cardiac arrhythmia by looking at the ECG signal with the naked eye. It is necessary to closely and comprehensively observe ECG records acquired from 12 channel electrodes to diagnose cardiac arrhythmias accurately.

In this study, cardiac arrhythmias in which abnormal signs are continuously observed in the ECG record were defined as 'persistent cardiac arrhythmias' (the meaning of 'persistent' in 'persistent cardiac arrhythmias' is different from that in 'persistent atrial fibrillation'). Representative persistent cardiac arrhythmias include atrial fibrillation (AF), first-degree atrioventricular block (I-AVB), left bundle branch block (LBBB), and right bundle branch block (RBBB). In the ECG signal of AF patients, an intact shape of the QRS wave is observed; however, because of the multiple irregular electrical simulations generated in the atrium, the P wave does not appear, and irregularly oscillating fibrillatory waves are observed^3^. In the case of I-AVB, the PR interval was continuously observed to be longer than 200 ms compared to that observed for a normal heart^2,4^. In the case of LBBB and RBBB, abnormal signs consistently occur, but they do not occcur on all of the 12 channels. In the LBBB condition, an M-shaped signal is often observed in the V6 channel, whereas in the RBBB condition, abnormal signs are observed in the V1 channel^5^. However, it is not easy to distinguish abnormal symptoms of persistent cardiac arrhythmia because of the presence of motion artifacts, noise due to contact with electrodes, and noise produced by the measuring device itself during the acquisition of the ECG signal.

Meanwhile, unlike persistent cardiac arrhythmias, cardiac arrhythmias that occur intermittently in normal signals are defined as "episodic cardiac arrhythmias." ST-segment elevation (STE) and ST-segment depression (STD), which are mainly caused by myocardial ischemia or infarction, are observed intermittently in the normal sinus rhythm (NSR). They are diagnosed when the height difference between the S-peak and the PR segment, which denotes the slope of the ST-segment of the ECG, is greater than ± 0.05 mV (one small scale of ECG is 1 mm, which means 0.04 s on the x-axis and 0.1 mV on the y-axis). The STD patient has an S-peak 3 mm lower than the PR segment in leads I, V5, V6, and aVL, and normal rhythms may appear in leads V2–V3^6^. On the other hand, in STE, an S-peak that is 4 mm higher than the PR segment is observed. The ECG signal of the ischemic ST-segment at V2–V4 is almost normal; however, in the non-ischemic ST-segment, abnormal symptoms are also observed at V2–V3^2^. Premature atrial contraction (PAC) occurs when the atrial tissue is temporarily excited above the threshold more quickly than the sinus node. It is a common symptom that 80% of people experience intermittently during their daily lives^6,7^. P-waves are not observed in the ECG signal of premature ventricular contraction (PVC) patients, and the heart rate is increased compared to that of a normal heart. In addition, the QRS width of PVC patients can increase by 120 to 210 ms compared to that of normal people, which is 60–80 ms^1,8^. However, it is difficult to accurately diagnose such episodic cardiac arrhythmias because their abnormalities are intermittently observed during normal heart rhythm.

Using ECG records, many research groups have proposed computer-aided diagnosis algorithms for diagnosing cardiac arrhythmias^3,7–9^. Inan et al. succeeded in classifying the PVC rhythm with 95.2% accuracy using a robust neural network after extracting the timing interval feature through wavelet transform to classify the beat of NSR and the abnormal rhythm due to PVC^8^. Shi et al. successfully detected the ST-segments in ECG signals using wavelet transform to diagnose STD and STE^10^. In the 2017 Physionet challenge, which distinguished between NSR, AF-induced signals, and other abnormal rhythms from ECG signals obtained from a short-single-lead ECG, Data et al. used short-time Fourier transform (STFT) to remove motion artifacts from ECG signals and achieved the highest classification performance (F1 score of 0.83) through multiple binary classifications^11^. In addition, Mehmood et al. classified cardiac arrhythmias through a two-dimensional (2D) ECG spectral image using STFT^9^. These algorithms classify cardiac arrhythmia by extracting the time–frequency features from a single ECG lead. However, as mentioned earlier, since some cardiac arrhythmias are observed only in a specific ECG channel, classification accuracy becomes reduced based on the type of cardiac arrhythmia to be classified.

Some studies have classified cardiac arrhythmias based on standard 12-lead ECG records. Baek et al. identified AF during NSR through a deep belief network based on a bidirectional LSTM network from clinically obtained 12-lead ECG records in a hospital^12^. Their algorithm detected AF during NSR with an F1 score of 75% for the internal validation dataset and 74% for the external validation dataset. Jadhav et al. classified 15 types of cardiac arrhythmia and NSR from 12-lead ECG signal with 86.67% accuracy using an artificial neural network-based system^13^. Ribeiro et al. classified six cardiac arrhythmias with F1 scores above 80% using deep neural networks composed of stacked transformations^14^. Mostayed et al. proposed a bidirectional LSTM network classifier to detect nine arrhythmias, including NSR, and achieved a final F1 score of 74.15^15^.

In the China Physiological Signal Challenge (CPSC) 2018, TM Chen et al. classified the nine classes of cardiac arrhythmias using a deep-learning neural network model. They suggested combining five 1D-CNN layers, a bidirectional gated recurrent unit, an attention layer, and dense output layer^16^. Then, they obtained 130 best validation models after tenfold cross-validation by training the model using a full 12-leads ECG records and 12 single-lead ECG records, respectively, and ensembled all of them. Finally, they can classify the nine classes of cardiac arrhythmia as high accuracy of the final F1 score from 0.82 to 0.84^16^. In the Computing in Cardiology Challenge 2020, Lin et al. proposed an explainable deep neural network using a class activation map^17^ to classify the 27 types of arrhythmias. Their model consisted of 12 1D-CNN layers with batch-normalization, a global average pooling layer that outputs the spatial average of the feature map of each unit of the last CNN layer, and a fully connected layer. Finally, they achieved the challenge score of 0.58^17^. In addition, Andrew Demonbreun and Grace M Mirsky automatically classified the 27 types of arrhythmias ECGs using wavelet analysis and a deep learning algorithm^18^. They used the SqueezeNet as the deep-learning model with a time–frequency feature map generated from wavelet transform. Then, they achieved challenge scores of 0.214 for the validation set and 0.205 for the full test set^18^.

The methods mentioned earlier require a complex training process for classifying arrhythmias. Because they trained those machine learning algorithms separately according to the 12 ECG lead positions, the 12 trained models need to be ensembled as one final model^16–18^. This study proposed a clustering algorithm to automatically extract abnormalities due to episodic cardiac arrhythmias, including persistent cardiac arrhythmia, from the short duration of the standard 12-lead ECG records and a deep learning algorithm to classify cardiac arrhythmias in patients accurately. The proposed clustering algorithm detects the signals having defects caused by arrhythmias, and from the clustered signal, we generated a 2D time–frequency feature map through the STFT. Finally, without additional ensemble processes, the proposed deep learning algorithm composed of a convolutional neural network (CNN) using a 2D time–frequency feature map classified the eight types of cardiac arrhythmia: AF, I-AVB, LBBB, RBBB, PAC, PVC, STD, and STE.

## Results

**Figure 1:**
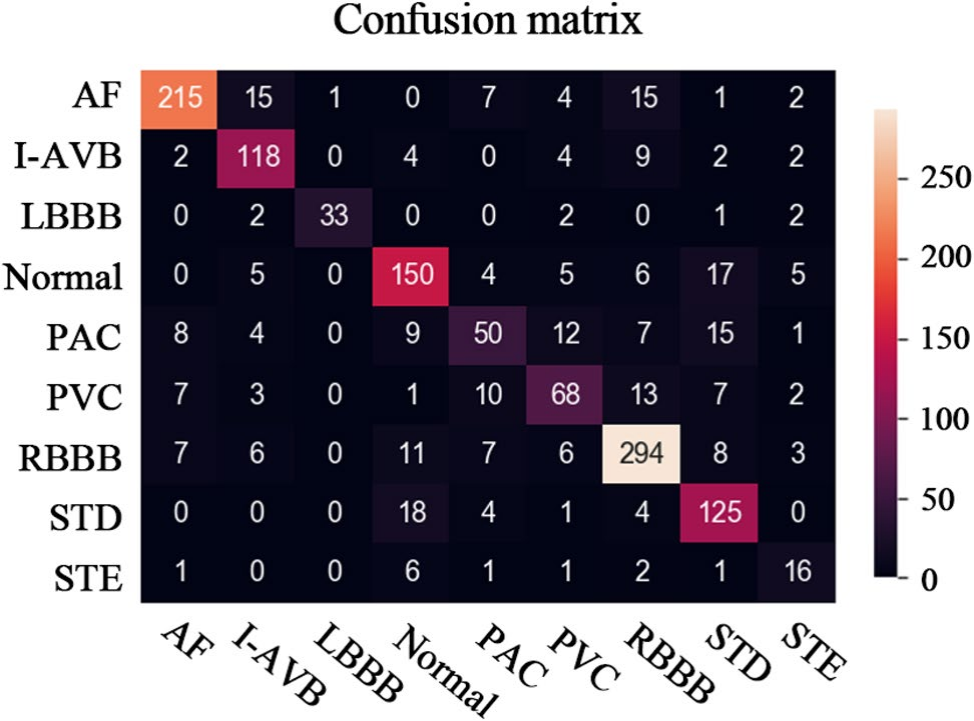
Confusion matrix for the proposed model.

**Figure 2:**
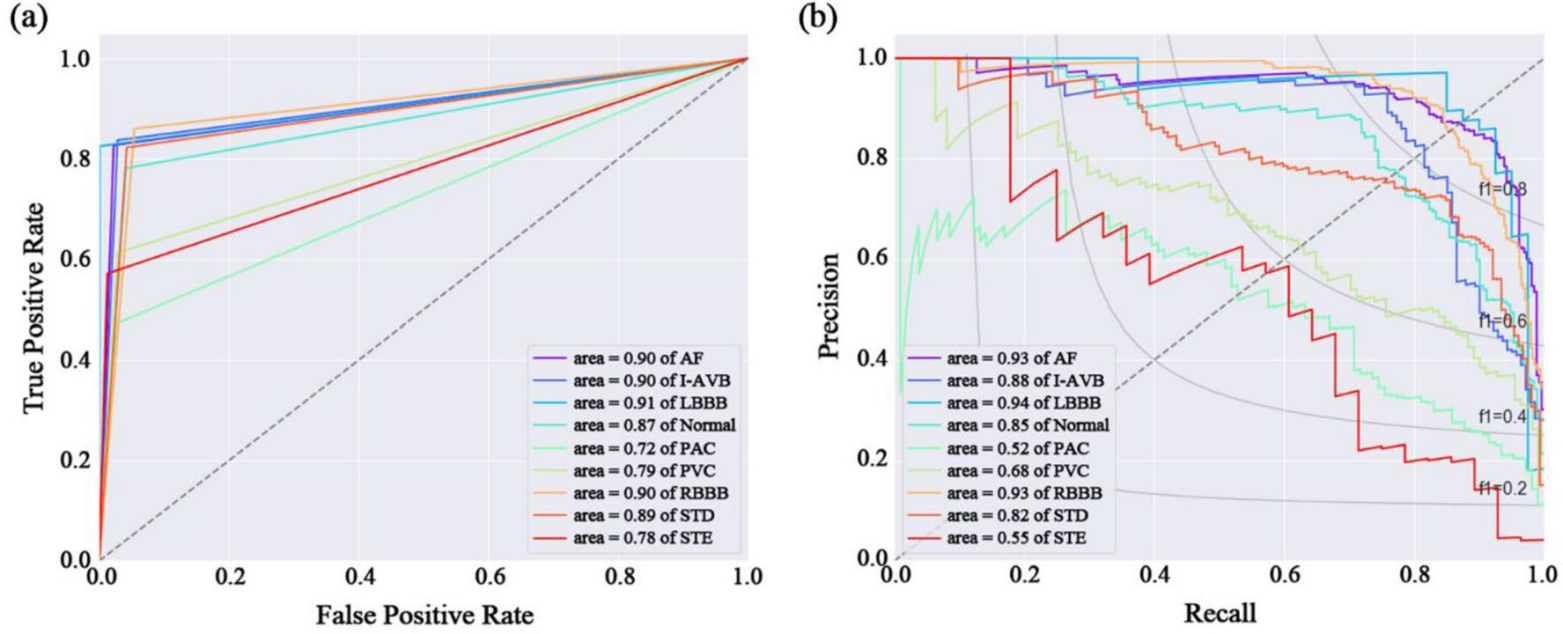
Performance curves for the proposed model. (**a**) Receiver operating characteristics (ROC) curves; (**b**) Precision-recall curves.

The results of classifying eight cardiac arrhythmias using the proposed algorithm are shown in Figs. 1, 2, and Table 1. The F1 scores of the proposed model were over 0.8 during the classification of persistent cardiac arrhythmias such as AF, I-AVB, LBBB, and RBBB. The highest F1 score was observed for LBBB prediction at 0.89, and the classified F1 scores for AF, RBBB, and I-AVB were 0.86, 0.85, and 0.80, respectively. On the other hand, the classification performance of episodic cardiac arrhythmias such as PAC, PVC, and STE was relatively low compared to persistent cardiac arrhythmias. The lowest F1 score (0.52) was observed during the classification of STE, which had the smallest number of data classes. The F1 scores for PAC and PVC were 0.53 and 0.64, respectively. In addition, the F1 scores for NSR and STD were moderate as scores of 0.77 and 0.76, respectively. There was a difference in the number of data classes used for training and testing. Accordingly, the final macro F1 score of the proposed model was 0.74; however, the weighted F1 score, considering the difference in the number of data classes, was 0.78.

**Table 1:** Summary of classification performance.

	Precision	Sensitivity	Specificity	F1 scores	AUC-ROC	AUC-PR
AF	0.90	0.83	0.98	0.86	0.90	0.93
I-AVB	0.77	0.84	0.97	0.80	0.90	0.88
LBBB	0.97	0.82	1.00	0.89	0.91	0.94
Normal	0.75	0.78	0.96	0.77	0.87	0.85
PAC	0.60	0.47	0.97	0.53	0.72	0.52
PVC	0.66	0.61	0.97	0.64	0.79	0.68
RBBB	0.84	0.86	0.95	0.85	0.90	0.93
STD	0.71	0.82	0.96	0.76	0.89	0.82
STE	0.48	0.57	0.99	0.52	0.78	0.55
Macro average	0.74	0.73	0.97	0.74	0.85	0.79
Weighted average	0.78	0.78	0.96	0.78	0.87	0.84

We used two performance curves to quantitatively evaluate the classification performance of the proposed model (Fig. 2). The area under the curves (AUCs) of the receiver operating characteristic (ROC) curves in the persistent cardiac arrhythmia classes were 0.90, 0.90, 0.91, and 0.90, respectively. In the episodic cardiac arrhythmia, the AUC for STD was the highest at 0.89, and the AUC for NSR was 0.89. The other episodic cardiac arrhythmia classes showed moderate accuracies of 0.72 for PAC, 079 for PVC, and 0.78 for STE, respectively (Fig. 2a). The macro average of AUC-ROC was 0.85, and the weighted average of AUC-ROC was 0.87 (Table 1).

The model's classification performance was evaluated using the precision-recall curve while taking the class imbalance into account. After adjusting for the ratio of the data classes, the classification accuracy showed high-performance with over 0.90 AUC for AF, LBBB, and RBBB among the persistent cardiac arrhythmia groups (AUCs: 0.93 AF, 0.94; I-AVB, 0.93; RBBB). The classification accuracies for I-AVB, NSR, and STD were over 0.80 (AUCs; 0.88 for I-AVB, 0.85 for NSR, and 0.82 for STD). However, as in the ROC curves, the AUCs for PAC, PVC, and STE were 0.52, 0.68, and 0.55, respectively, showing a relatively low classification accuracy (Fig. 2b). The macro average of AUC-PR was 0.79, and the weighted average of AUC-PR was 0.84 (Table 1).

## Discussion

We proposed an automatic feature extraction algorithm and a CNN classification model to classify nine types of cardiac arrhythmias, including NSR. The main findings of this study are as follows:

1. The F1 score of the proposed model for arrhythmia classification was 0.78, which was calculated considering the variable number of classes.
2. In the performance curves, the AUC of the proposed model was over 0.90 in the classification of persistent cardiac arrhythmias (AF, LBBB, and RBBB). It means that our proposed model is more suitable for classifying persistent cardiac arrhythmias than for the classification of episodic cardiac arrhythmias.

Some studies classified the cardiac arrhythmias using the standard 12-lead ECG signals and the time–frequency feature maps generated from STFT or wavelet tranform^16–18^. They usually used long enough signals to extract features with the diseases-specific markers by persistent or episodic arrhythmias. Therefore, they did not need to classify the ECG signals according to the two types of arrhythmias. However, we used only three cycles of ECG signals, which is around from 2 to 3 s. In persistent arrhythmias, since the abnormalities continuously occur over time, the disease-specific marker can be captured easily, even in a short time ECG signals. On the other hand, in the case of episodic arrhythmias, the disease-specific points come out occasionally over time, which is difficult to detect in a short ECG record. Accordingly, we proposed the clustering algorithm to find the short ECG sequence likely having the disease-specific abnormality.

In this study, the STFT of ECG records containing 2–3 PQRST waves was performed to detect the change in the dominant frequency with time. In the ECG waveform, the dominant frequency with the most prominent spectral power density can vary depending on the presence of heart disease, and abnormal symptoms due to cardiac arrhythmias can also be traced through frequency changes^19–22^. These abnormal signs due to cardiac arrhythmia could be observed in the ECG signals extracted through the clustering algorithm proposed in this study. These were reflected in the time–frequency map generated during the STFT (Supplementary Figures S1–S11).

In AF patients, irregular waveforms are captured in the P-wave of the ECG, and the frequency during AF ranges from 5.3 to 10 Hz^23^. It was also observed in the time–frequency map of the proposed algorithm in this study (Supplementary Figure S4). Our results were similar to those of Stridh et al.^21^. Furthermore, the change in the waveform due to LBBB and RBBB was captured in the ECG waveforms of leads I, V5, and V6 (LBBB), and leads III, aVL, and aVF (RBBB) and their time–frequency feature maps (Supplementary Figures S11–S14). These results are consistent with Benmalek et al.^24^, whose study involved time–frequency spectrogram analysis.

PAC patients have an abnormal P-wave shape, which denotes the electrical depolarization of the atrium. Sometimes, this odd P-wave shape is partially or wholly buried in the T-wave due to the electrical repolarization of the ventricles. However, in most PAC patients, either the interval between the P-wave and the QRS wave is slightly longer than that in the normal ECG or the P-wave, and the QRS wave appears similar to the sinus rhythm, making it difficult to distinguish it from that of a normal heart^6^. These forms can also be confirmed in the ECG signals selected in this study using the proposed clustering algorithm (Supplementary Figure S7). On the other hand, the rise and fall of ST-segment by STD and STE were captured in V5 and V6, and a more pronounced difference was observed in the temporal frequency maps of V5 and V6 (Supplementary Figures S15–S18).

In the standard 12-lead ECG records used in this study, multiple labels ECG records were acquired from patients with two or more cardiac arrhythmias (Supplementary Table S1)^25^. Some PAC patients had abnormalities due to STD and STE, and in the signals of LBBB patients, the RBBB and AF signals were observed simultaneously. Accordingly, in situations where our proposed clustering algorithm was unable to extract the signal of the target arrhythmia but was able to extract the signal due to the complication as a representative signal, the error of the arrhythmia classification was able to increase. Furthermore, the classification error might have increased since the noisy signal was extracted as a representative signal rather than the most obvious signal in the NSR (Supplementary Figure S1). Nevertheless, the accuracy during the classification of persistent cardiac arrhythmias, such as AF, LBBB, and RBBB, was high.

In this study, the number of ECG records for each arrhythmia group was unbalanced; the arrhythmias with the most records was RBBB (1675), excluding multiple arrhythmia classes. The number of STE classes was the lowest at 185. This class imbalance problem was present not only in the testing set but also in the training set. In the testing set, the arrhythmia group with the highest number of ECG records was AF (260), and the arrhythmia group with the least number of ECG records was STE (28). This class imbalance was also expected to affect the classification performance of the proposed model^26^.

Accordingly, we evaluated it using the ROC curve, precision-recall curve, and weighted F1 score to prevent the under-or over-estimation of the proposed model due to class imbalance^26^. The ROC curve shows the true positive rate according to the classified data's false positive rate, and each class's classification accuracy can be evaluated using the AUC. That is, the ROC does not consider the difference in the number of ECG records between arrhythmias. However, the precision-recall curve allows us to check the classification performance considering the difference in the number of ECG records^26,27^. In addition, when calculating the final F1 score, the model was objectively evaluated by multiplying the F1 score by the weight, taking into account the number of ECG records for each class during the classification of multiclass data.

Our proposed model has some limitations. First, even though our proposed model used the same ECG records offered in the CPSC 2018, we did not test our proposed model using the hidden ECG records used for the public validation in the Challenge. Accordingly, we were not able to compare the performances objectively with those of the previous algorithms. Second, some ECG records have multiple labels of cardiac arrhythmias. However, in this study, we considered the first diagnosis as the representative cardiac arrhythmia class. Third, the 2D feature map we proposed was interpolated to match the size of images used in the input of the 2D CNN model. Even though interpolating the STFT images may not be affected the meaning of the original ECG signal^9^, it might have affected the minor defects caused by the episodic arrhythmias. In addition, the 2D time–frequency feature map was generated by stacking the extracted feature maps of 12 leads ECG signals from 0 to 50 Hz. Therefore, it may be challenging to classify cardiac arrhythmias with signal fault above 50 Hz accurately. However, the 2D time–frequency feature map we used in this study reduced the complexity of the model by reducing the number of parameters to be trained.

This study proposed a clustering algorithm that can classify persistent cardiac arrhythmia and episodic cardiac arrhythmias using standard 12-lead ECG records. The 2D CNN model also used time–frequency feature maps to classify the eight types of arrhythmias without the complex ensemble process. In particular, the proposed algorithm showed high performance in the classification of persistent cardiac arrhythmias. The somewhat low accuracy in classifying episodic arrhythmias may be due to class imbalance in the episodic arrhythmia group and no consideration of multilabel records. Thus, for future study, we will implement a deep learning model that can classify the multilabel ECG records with more ECG records to improve and verify the robustness of the model. Suppose this problem is compensated for through learning and verification using more ECG records for episodic arrhythmias. In that case, we believe our clustering algorithm can be used as an auxiliary device for diagnosing cardiac arrhythmias. The proposed methods can help physicians and experts in related fields diagnose accurate cardiac arrhythmias by referring to the automatic classification results of ECG records.

## Methods

### Standard 12-leads ECG records

In this study, the standard 12-leads ECG records provided by the CPSC 2018 were used; it consists of 6877 ECG records (male: 3699, female: 3178)^28,29^. These ECG records were obtained from eight types of cardiac arrhythmia patients (AF, I-AVB, LBBB, RBBB, PAC, PVC, STD, and STE patients), including NSR signals without any cardiac arrhythmias. ECG records were measured for a minimum of 6 s to a maximum of 60 s at a sampling frequency of 500 Hz.

### Preprocessing

#### Removing motion artifact and baseline wandering

Figure 4 shows the overall process used by the proposed artificial intelligence algorithm to classify the eight types of cardiac arrhythmias. First, we applied a band-pass filter to remove the noise and baseline wandering noise while taking measurements using an ECG device. In this study, we filtered the signals between 0.5 and 50 Hz to remove motion artifacts and minimize data loss based on the advanced research done by Stridth et al.^21^.

#### R-R grouping algorithm

The filtered 12-lead ECG signals were grouped based on the R-peaks detected from the filtered lead I ECG record. This preparation process for generating the sequence signal includes the change in heart rate and shape deformation due to the arrhythmias to classify the eight cardiac arrhythmias. The R-peak of each ECG signal was extracted by combining the Pan and Tompkins algorithm and a robust thresholding algorithm (refer to the Supplementary material)^30,31^. Because the squaring process in the Pan and Tompkins algorithm buries some of the QRS complexes having too narrow or too wide QRS width, we used the combined algorithm using the Shannon energy.

#### Clustering algorithm for extracting representative signals

**Figure 3:**
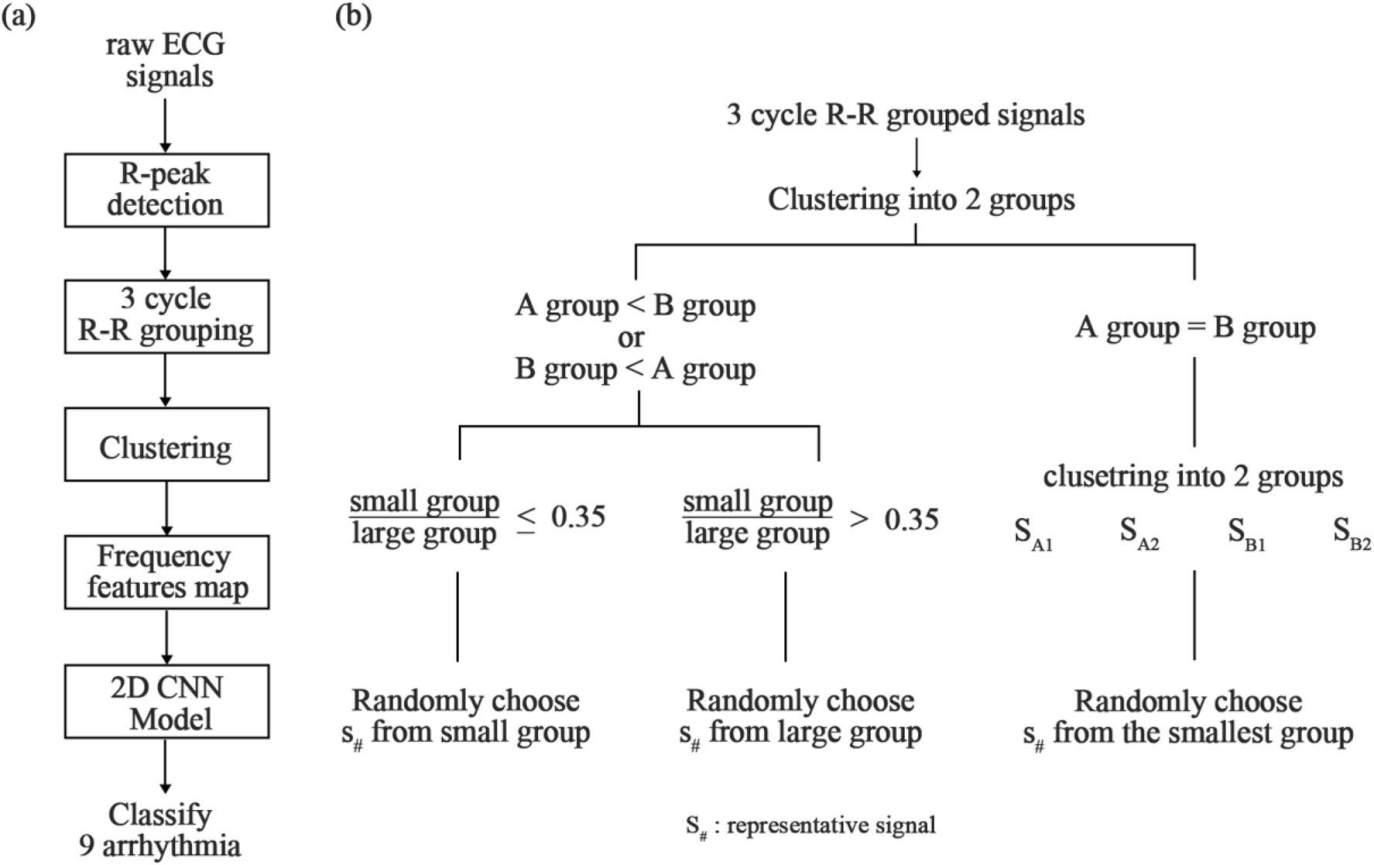
The workflow of the proposed model. (**a**) The workflow for the entire process; (**b**) the workflow for the clustering algorithm.

A clustering algorithm for extracting representative signals having geometric information due to episodic arrhythmias is shown in the workflow diagram in Fig. 3. First, the clustering algorithm divides continuous signal sequences with three PQRST signals into two groups (A and B groups). Then, our algorithm randomly extracts the representative signal from the small groups when the ratio of the small group to the large group is 0.35 or less to find a group of signals having abnormal signs due to a single cardiac arrhythmia. If this ratio is greater than 0.35, the representative signal is randomly extracted from the large group. Here, this threshold value of 0.35 was fine-tuned empirically from the minimum threshold of 0.3 when the clustering algorithm selected one R-R series among four R-R series. If the number of groups is the same, clustering is performed again in each group, and each group is subdivided into two groups; S_A1_ and S_A2_ groups are grouped from group A, then S_B1_ and S_B2_ groups are from group B. The representative signal is then extracted from the smallest group. The extracted signal, which comprises abnormal signs due to episodic and persistent cardiac arrhythmias, was used as a representative signal (S_#_).

In this study, we used the K-means clustering function of the Scikit-learn library to group R-R sequence signals based on the distance information between the data points of each signal. The classical K-means algorithm generates the clusters according to the parameter K^32^. This study divided the R-R sequences into two groups by setting the parameter K as 2, which controls the number of centroids. The classical K-means algorithm randomly selects the central points and allocates data to the nearest group by measuring the Euclidian space between centroids and data points. Then, it updates the centroid until the Euclidian distance closes to zero. Therefore, it is highly dependent on the initialization of the centroids and spends many memories. To address these problems of sensitivity and efficiency, we used the centroid initialization algorithm suggested by Arthur and Vassilvitskii^33^; it initializes the centroids to be generally distant from each other. Furthermore, in updating the centroids, we used the triangle inequality suggested by Elkan to accelerate the K-means algorithm by avoiding unnecessary distance calculations^34^.

#### Time–frequency feature map extraction

**Figure 4:**
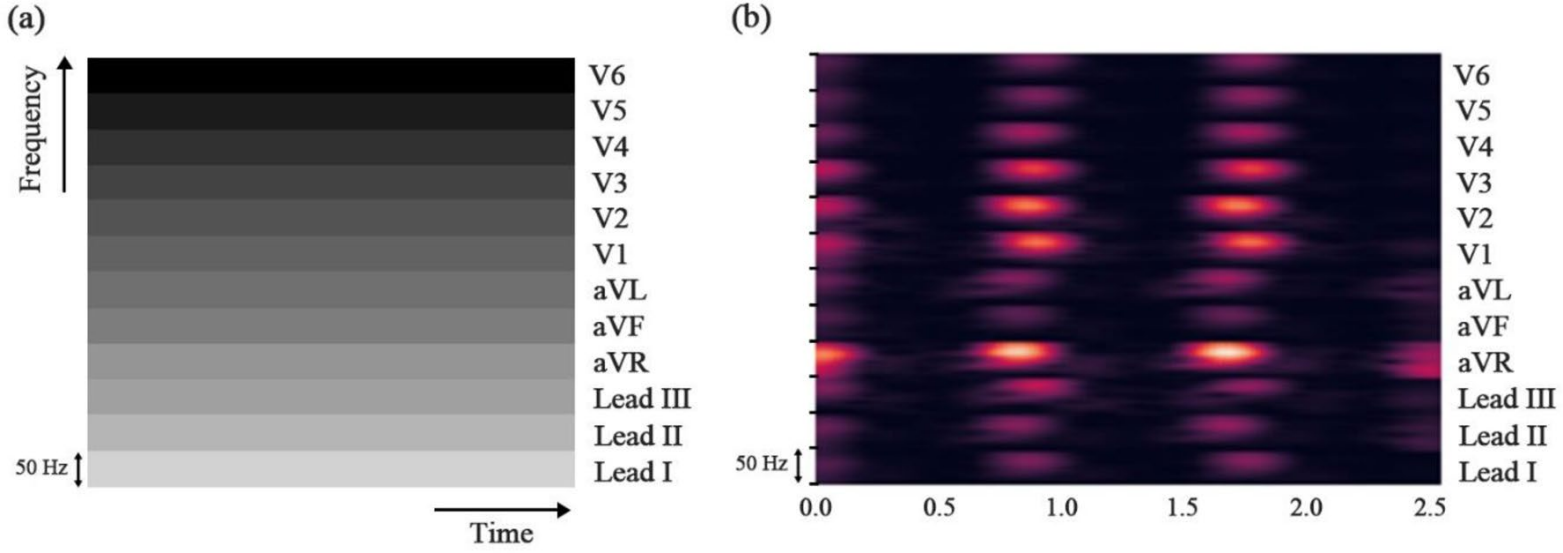
Time–frequency feature map. (**a**) Mimetic diagram of the time–frequency feature map; (**b**) a time–frequency feature map generated from the proposed algorithm.

The representative signals extracted from each of the 12 channels were transformed into a time–frequency feature map through STFT. The ECG records generally contain meaningful information in the frequency band between 0 and 150 Hz^35^; however, in this study, we used only information in the frequency band between 0 and 50 Hz generated from STFT (frequency characteristics due to AF are captured in the 2.5–25 Hz frequency band^21^). The time–frequency feature maps extracted from the 12 ECG channels were sequentially merged and used (Fig. 4a). For the STFT, we used the signal toolkit of the Scipy library. Accordingly, the discrete-time STFT of given time-domain signals *x[n]* was expressed with window sequence w[n] as follows.

1 $$STFT\left\{x[n]\right\}(m, \omega )=X\left(m, \omega \right)= \sum_{n=-\infty }^{\infty }x[n]w[n-m]{exp}^{-j\omega n}$$

Here, n is the time variable that controls the temporal resolution and was set to 600 ms (300 samples per segment). m is the overlap window size, which is decided according to the fixed time frame and the overlap window's hop size. In this study, the time frame was 0.002 s (sampling frequency of 500 Hz), and the window's hop size was set as 270 samples. Accordingly, m was decided as 540 ms to minimize the decrease in time resolution due to the increase in frequency resolution.$\omega$ is the frequency variable and determined as 1.667 Hz according to the trade-off relationship with temporal resolution. As we mentioned above, the STFT spectrum in each ECG channel was extracted from 0 to 50 Hz and stacked vertically as shown in Fig. 4a. Because the length of the R-R sequences differs according to the type of arrhythmia, the time–frequency maps were transformed into final time–frequency maps with a shape of $120\times 120$, using linear interpolation^9^ (Fig. 4b). Interpolating or augmenting a 1D ECG signal itself can distort the signal's meaning, but interpolation or augmentation of a 2D STFT image may minimize the effect on the original 1D signal^9^. Finally, these time–frequency maps were used as inputs to the 2D CNN model.

### Model structure and training

**Figure 5:**
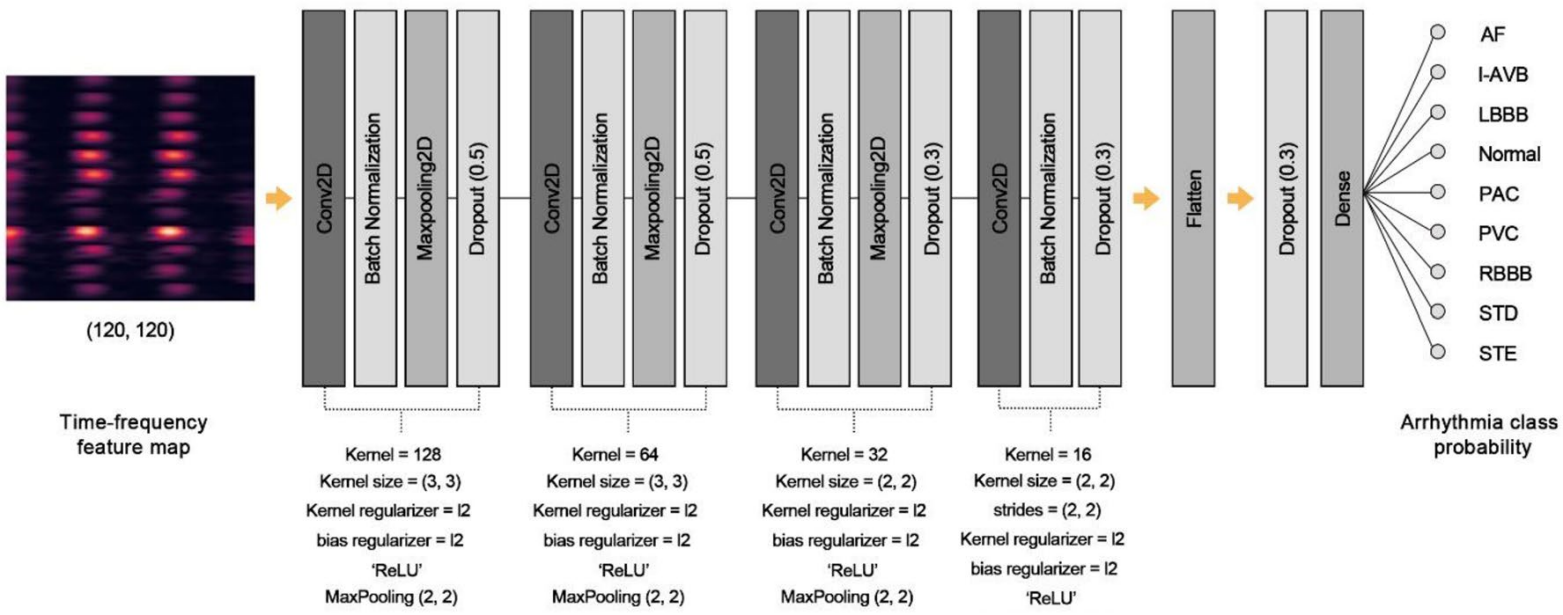
The structure of the 2D CNN model.

The CNN model for classifying cardiac arrhythmias consisted of four 2D CNN layer groups; the first, second, and third groups consisted of 2D CNN layers, a BatchNormalization (BN) layer, a 2D Maxpooling (2DmaxP) layer, and Dropout (2DCNN-BN-2DmaxP-Dropout), and the last group consisted of 2DCNN-BNDropout. Figure 5 shows the configuration of our proposed model in detail. The first and second 2D CNN layers had 128 and 64 kernels of size (3, 3). The third and fourth 2D CNN layers consisted of 32 and 16 kernels of size (2, 2). The kernel of the fourth 2D CNN layer moves with strides (2, 2), while others move with (1, 1). The pooling size of every 2DmaxP was (2, 2). A dropout rate of 0.5 was applied to the first and second 2DCNN-BN-2DmaxP groups and 0.3 for the third and last groups. The pooling size of every 2DmaxP was (2, 2). We applied the kernel and bias regularizers to each 2D CNN layer with the l2 loss lambda of 0.00015 to prevent the model from overfitting with the training set. The outputs of four 2D CNN layer groups are fed into the Dense layer by flattening as the 1D array to classify eight types of arrhythmias. ReLU was used as the activation function for each layer, and "softmax" was used for the output layer^36^. We decided on this model structure by trials to find the hyperparameter with the best performance (see Supplementary Table S2 for details).

80% of the ECG records were used for training (5484) and 20% for testing (1372). The model was trained 500 times using the sparse categorical cross-entropy loss function and the Adam optimization function with a learning rate of 0.01^37^. The optimal model with hyperparameters was found using fivefold cross-validation. For the fivefold cross-validation, the whole training set was randomly separated into five groups, then used four groups for training and the remaining one group for validation. Here, the remaining group was changed according to each fold. The classification performance of the final model was evaluated in terms of accuracy, recall, and precision. The macro F1 score, weighted F1 score, confusion matrix, ROC curve, and precision-recall curve were also evaluated^27^. Here, the macro F1 score and weighted F1 score were calculated by considering the different numbers of the data class.

2 $$\text{macro} \; \text{F}1 \; \text{ score}= \frac{\sum {F1}_{arrhythmia \; class}}{number \; of \; arrhythmia \; classes}$$

3 $$\text{weighted} \; \text{F}1\; \text{ score}=\sum {F1}_{class}{W}_{class}$$

4 $${W}_{class}=\frac{number \; of \; data \; in \; \# \; class}{total \; number \; of \; dataset}$$

## Supplementary Information

Supplementary Information.

## Data Availability

The standard 12-lead ECG records used in this study can be downloaded from the Physionet website for Classification of 12-lead ECGs: the PhysionNet–Computing in Cardiology Challenge 2020; https://physionet.org/content/challenge-2020/1.0.1/. All results were generated through machine learning performed by the authors based on the methods described in the text.
