# Supplementary material for: Convolutional neural network for classification of eight types of arrhythmia using 2D time–frequency feature map from standard 12-lead electrocardiogram
Source: Sci Rep. 2021 Oct 14;11:20396. doi: 10.1038/s41598-021-99975-6 (PMC8516863; doi:10.1038/s41598-021-99975-6)
Supplement: Supplementary file 1 — Supplementary Information. [file 41598_2021_99975_MOESM1_ESM.pdf]

Convolutional Neural Network for Classification of Eight types of Arrhythmia using 2D Time-Frequency Feature Map from Standard 12-lead Electrocardiogram

Da Un Jeong[1], Ki Moo Lim[1, 2*]

[1]Kumoh National Institute of Technology, IT convergence engineering, Gumi, 39253, Republic of Korea.
[2]Kumoh National Institute of Technology, Medical IT convergence engineering, Gumi, 39253, Republic of Korea.
+82-54-478-7780
dawny6960@kumoh.ac.kr
[*]kmlim@kumoh.ac.kr

Supplementary Materials

1. R peak detection algorithm for R-R grouping

We detected the R-peak of each ECG signal by combining the Pan and Tompkins algorithm and a robust thresholding algorithm. First, we filtered the raw ECG signal at 2 to 50 Hz with the Butterworth to remove noise and enhance the QRS complex and calculated the slope by differentiating the filtered signal (d[n]). Then, the Shannon Energy was computed by the nonlinear transformation that is consisted of squaring the derivate to get the energy values (e[n]) and applying the robust thresholding function (e_{th}[n]).

$$\mathrm{e}[\mathrm{n}] = d^2[n] \tag{1}$$

$$e_{th}[n] = \begin{cases} 0, & e[n] < \eta \\ e[n], & otherwise \end{cases} \tag{2}$$

$$\eta = 0.5 \times \sigma_e \tag{3}$$

In above equations, η is a threshold parameter. In this study, we set η as half the standard deviation (σ_e) of the energy values. The squaring process buries some of QRS complexes having too narrow or too wide QRS width. Accordingly, we used the Shannon enery to detect the R-peak. The Shannon energy (s[n]) was calculated from the normalized threshold energy signal ($\tilde{e}_{th}[n]$).

$$\tilde{e}_{th}[n] = \frac{e_{th}[n]}{max_{n=1}^{N}(|e_{th}[n]|)} \tag{4}$$

$$\mathrm{s}[\mathrm{n}] = -\tilde{e}_{th}{}^2[n] \log \tilde{e}_{th}{}^2[n] \tag{5}$$

Then, the Shannon energy was smoothed using the finite impulse response (FIR) filter to reduce the effect of multiple peaks within the QRS complex and to generate peaks corresponding to the QRS-complex portions. The R-peak was detected using the first-order Gaussian differentiator operation and the zero crossings algorithms. The first-order Gaussian differentiator operator found the locations of candidate R-peaks from the Shannon energy signals by using follows equations.

$$w_d[m] = w[m+1] - w[m] \quad (6)$$

$$\mathrm{w[m]} = e^{-\frac{1}{2}\frac{\left(m-\frac{M}{2}\right)^2}{\sigma^2}} \quad (7)$$

here, w[m] is the M-point Gaussian window. For detecting the candidate R-peaks in the Shannon energy signal, the convolution operation was applied on the Shannon energy signal and the first-order Gaussian differentiator sequence ($w_d[m]$) as shown in equation (8), and from this equation, the zero-crossing function (z[n]) was output.

$$\mathrm{z[n]} = \sum_{k=-\infty}^{\infty} w_d[k]s[n-k] \quad (8)$$

The zero-crossing function has both positive and negative zero-crossing points because the first-order Gaussian differentiator has the anti-symmetric nature. Accordingly, the positive zero-crossing was defined as the zero-crossing point with a positive slope, in which z[n] moves from positive value to negative value, and the negative zero-crossing was defined as the zero-crossing point with a negative slope, in which z[n] moves inversely from negative value to positive value. Because the negative zero-crossing points indicate the location of the peak in the Shannon energy signal, which is associated with the R-peak of the original ECG signal, we finally detected the R-peak by finding the negative zero-crossing points in z[n].

Supplementary Tables

Supplementary Table S 1. The number of arrhythmia classes

	AF	I-AVB	LBBB	Normal	PAC	PVC	RBBB	STD	STE
**AF**	1221	-	-	-	-	-	-	-	-
**I-AVB**	0	722	-	-	-	-	-	-	-
**LBBB**	29	8	199	-	-	-	-	-	-
**Normal**	0	0	0	918	-	-	-	-	-
**PAC**	4	3	10	0	544	-	-	-	-
**PVC**	8	5	6	0	3	627	-	-	-
**RBBB**	172	10	0	0	55	51	1675	-	-
**STD**	30	6	0	0	6	18	20	786	-
**STE**	2	4	4	0	2	2	16	2	185

* AF, atrial fibrillation; I-AVB, first-degree atrioventricular block; LBBB, left bundle branch block; Normal, Normal sinus rhythm; PAC, premature atrial contraction; PVC, premature ventricular contraction; RBBB, right bundle branch block; STD, ST-segment depression; STE, ST-segment elevation

Supplementary Table S 2. The performance comparison for the decision of model configuration and hyperparameter tuning

No.	Model structure		Accuracy	Macro F1	Weighted F1
1	Input Layer	Input shape = (120, 120)	0.72	0.63	0.71
	2D CNN	128 @ (3, 3)			
	2D CNN	64 @ (3, 3)			
	2D CNN	32 @ (2, 2)			
	2D CNN	16 @ (2, 2)			
	Flatten				
	Dropout	dropout rate = 0.3			
	Dense	nueron = 20			
	Output Layer	nueron = 9			
2	Input Layer	Input shape = (120, 120)	0.73	0.64	0.72
	2D CNN -BN	128 @ (3, 3)			
	2D CNN	64 @ (3, 3)			
	2D CNN	32 @ (2, 2)			
	2D CNN-BN	16 @ (2, 2)			
	Flatten				
	Dropout	dropout rate = 0.3			
	Dense	nueron = 20			
	Output Layer	nueron = 9			
3	Input Layer	Input shape = (120, 120)	0.74	0.68	0.74
	2D CNN – BN–2DmaxP	128 @ (3, 3)			
	2D CNN – BN–2DmaxP	64 @ (3, 3)			
	2D CNN – BN–2DmaxP	32 @ (2, 2)			
	2D CNN – BN–2DmaxP	16 @ (2, 2)			
	Flatten				
	Dropout	dropout rate = 0.3			
	Output Layer	nueron = 9			
4	Input Layer	Input shape = (120, 120)	0.76	0.71	0.76
	2D CNN – BN–2DmaxP	128 @ (3, 3)			
	2D CNN – BN–2DmaxP	64 @ (3, 3)			
	2D CNN – BN–2DmaxP	32 @ (2, 2)			
	2D CNN – BN	16 @ (2, 2)			
	Flatten				
	Dropout	dropout rate = 0.3			
	Output Layer	nueron = 9			
5	Input Layer	Input shape = (120, 120)	0.77	0.72	0.77
	2D CNN – BN–2DmaxP-drop	128 @ (3, 3), dropout rate = 0.3			
	2D CNN – BN–2DmaxP-drop	64 @ (3, 3), dropout rate = 0.3			
	2D CNN – BN–2DmaxP-drop	32 @ (2, 2), dropout rate = 0.3			
	2D CNN–BN-drop	16 @ (2, 2), dropout rate = 0.3			
	Flatten				
	Dropout	dropout rate = 0.3			
	Output Layer	nueron = 9			
6	Input Layer	Input shape = (120, 120)	0.77	0.73	0.75
	2D CNN – BN–2DmaxP-drop	128 @ (3, 3), dropout rate = 0.5			
	2D CNN – BN–2DmaxP-drop	64 @ (3, 3), dropout rate = 0.5			
	2D CNN – BN–2DmaxP-drop	32 @ (2, 2), dropout rate = 0.3			
	2D CNN–BN-drop	16 @ (2, 2), dropout rate = 0.3			
	Flatten				
	Dropout	dropout rate = 0.3			
	Output Layer	nueron = 9			
7*	Input Layer	Input shape = (120, 120)	0.78	0.74	0.78
	2D CNN – BN–2DmaxP-drop	128 @ (3, 3), dropout rate = 0.5, L2 regularization			
	2D CNN – BN–2DmaxP-drop	64 @ (3, 3), dropout rate = 0.5, L2 regularization			
	2D CNN – BN–2DmaxP-drop	32 @ (2, 2), dropout rate = 0.3, L2 regularization			
	2D CNN–BN- Dropout	16 @ (2, 2), dropout rate = 0.3, L2 regularization			
	Flatten				
	Dropout	dropout rate = 0.3			
	Output Layer	nueron = 9			

* model no.7 is our proposed model; 2D CNN, 2-dimensional convolution neural network layer; BN, Batch normalization layer; 2DmaxP, 2-dimensional maxpooling layer

Supplementary Figures

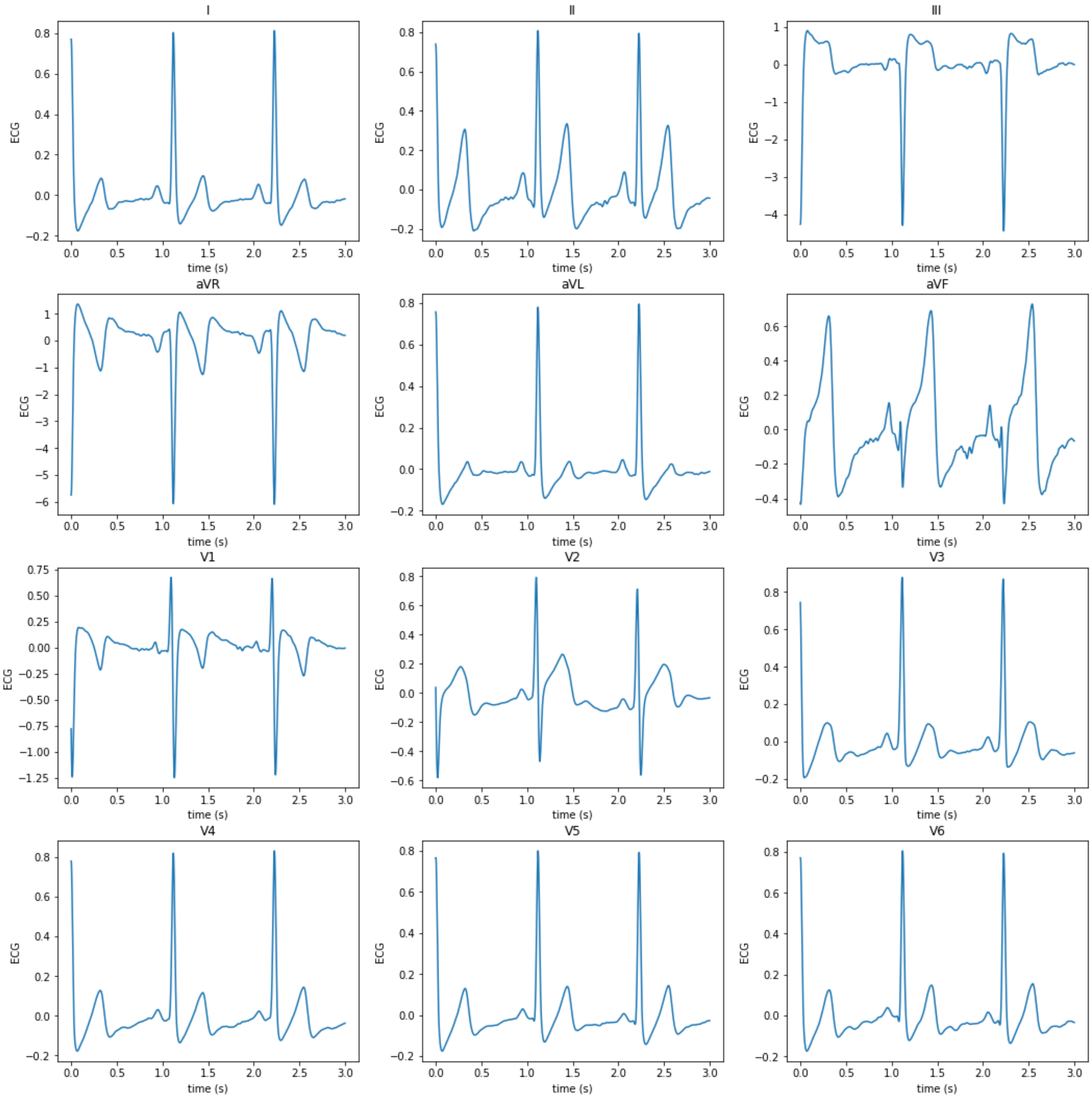

Supplementary Figure S 1. ECG waveform of normal patient.

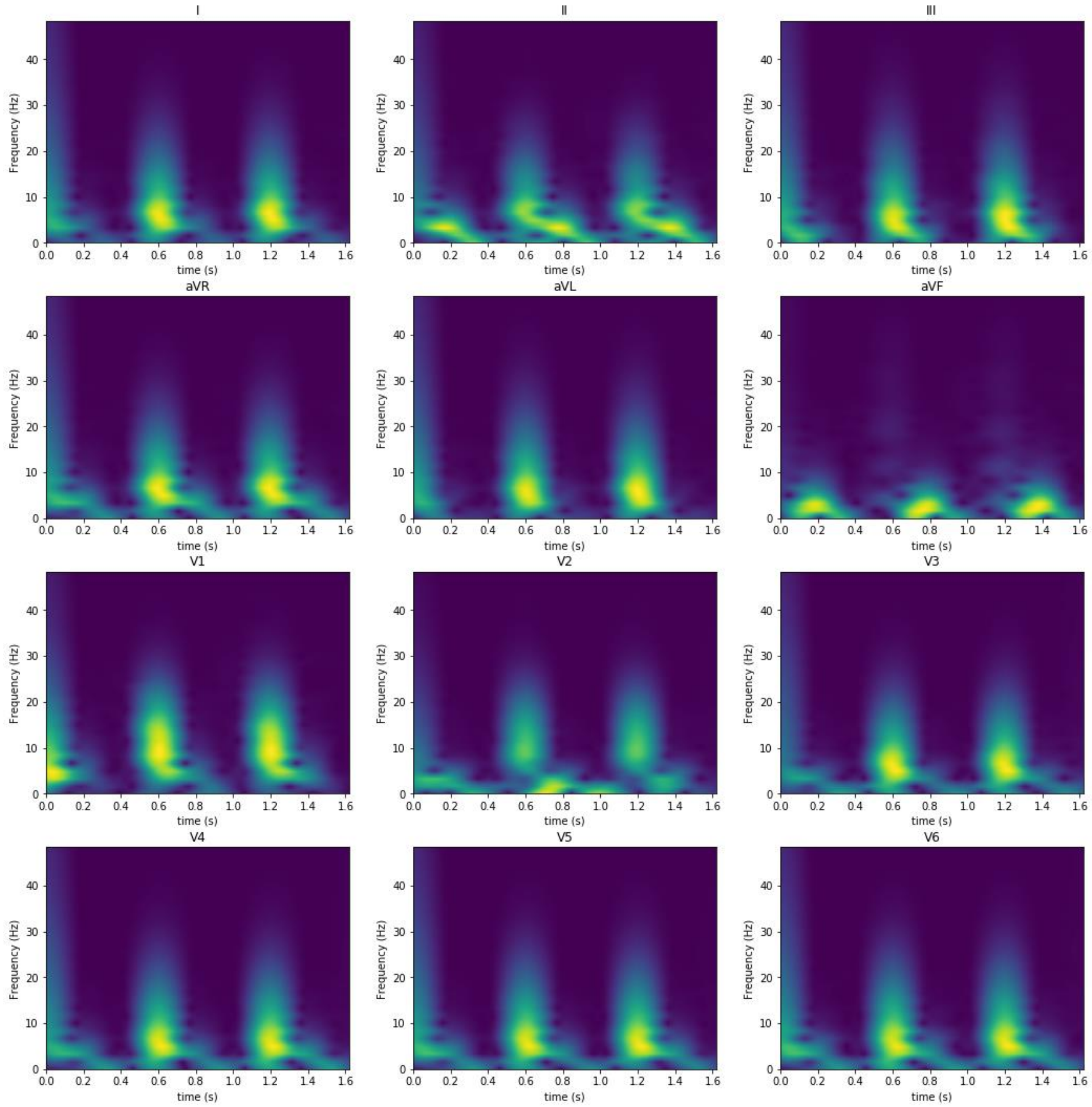

Supplementary Figure S 2. Time-frequency maps of Normal patient.

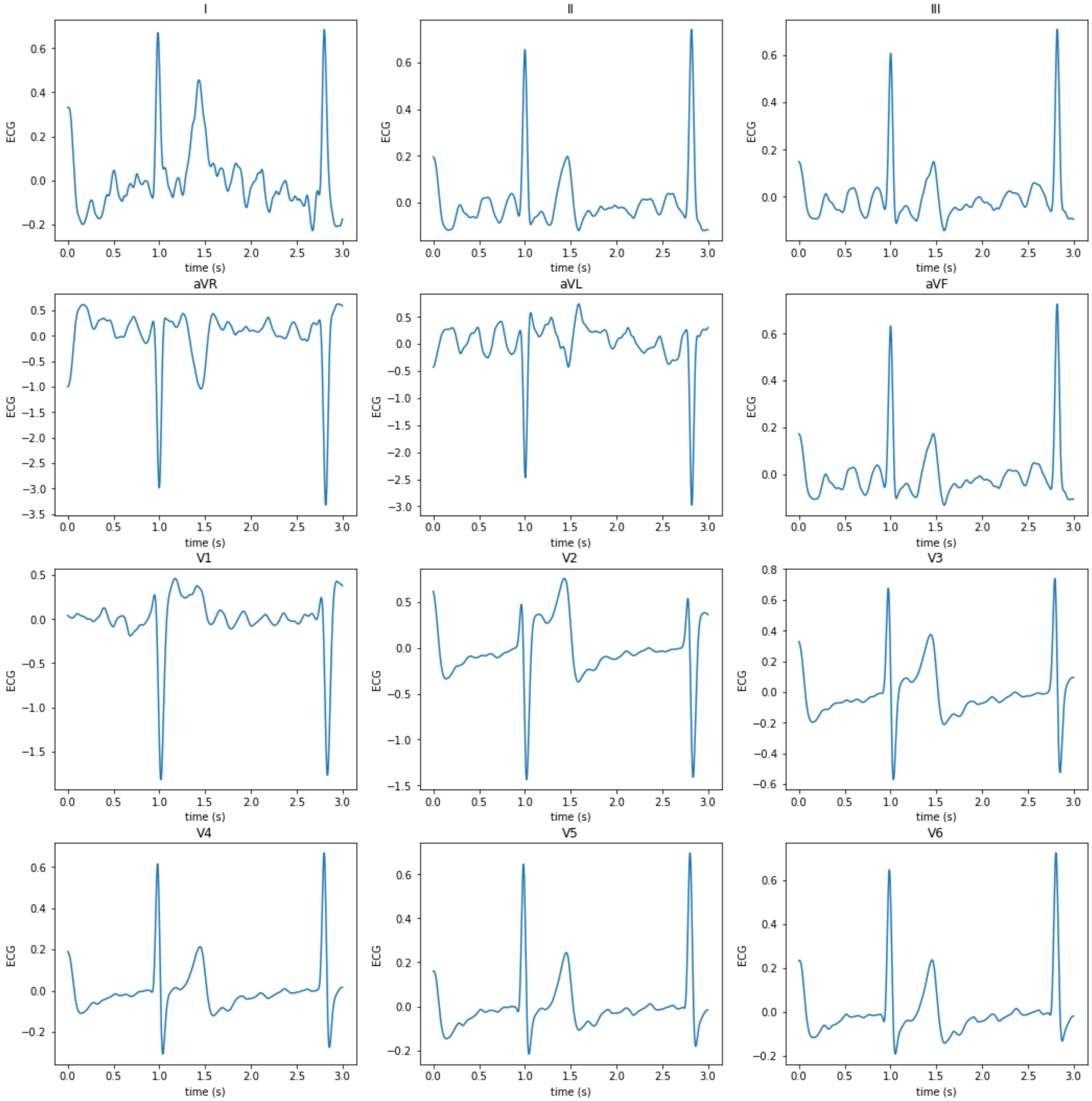

Supplementary Figure S 3. ECG waveform of AF patient

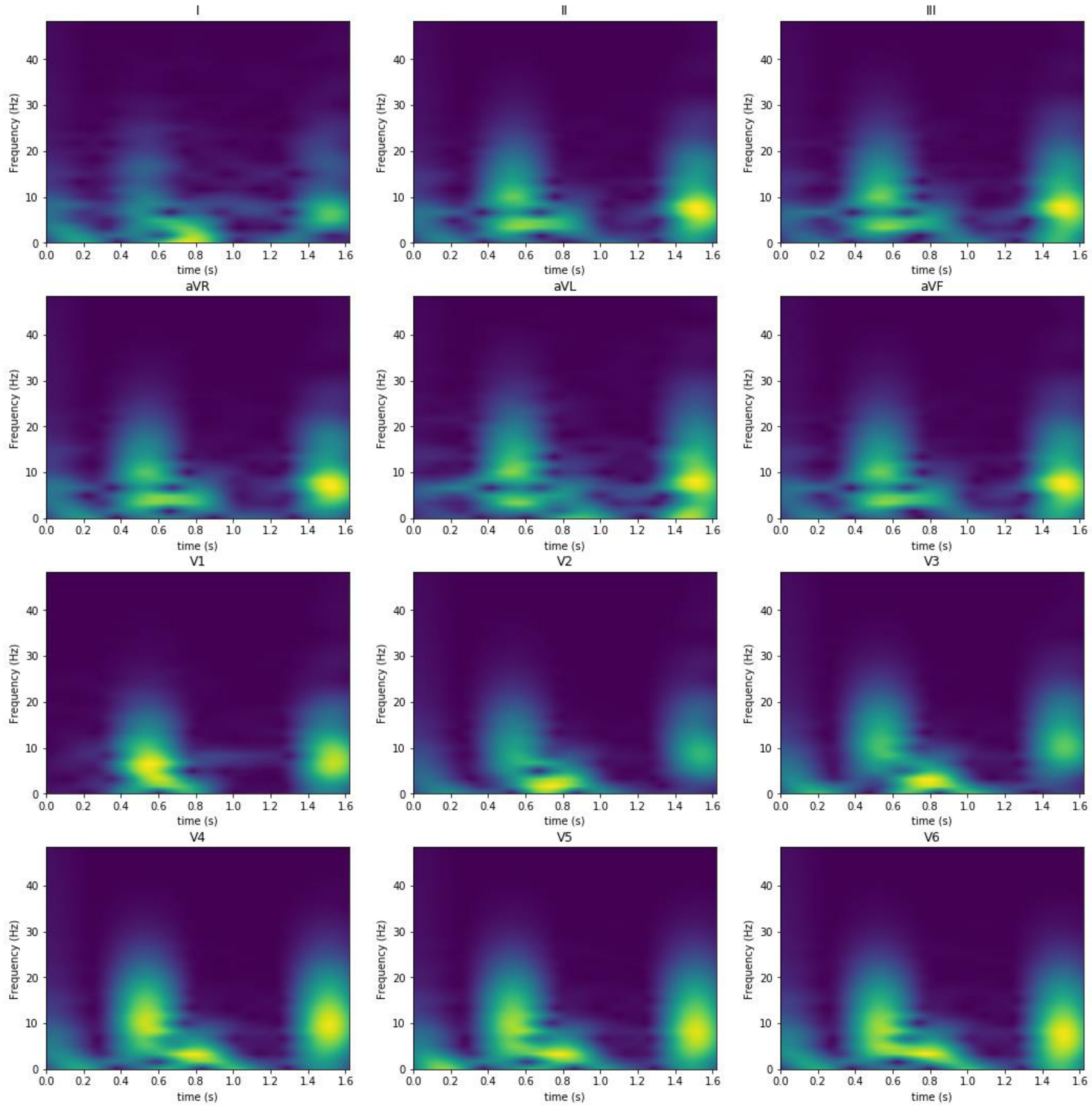

Supplementary Figure S 4. Time-frequency map for AF patient

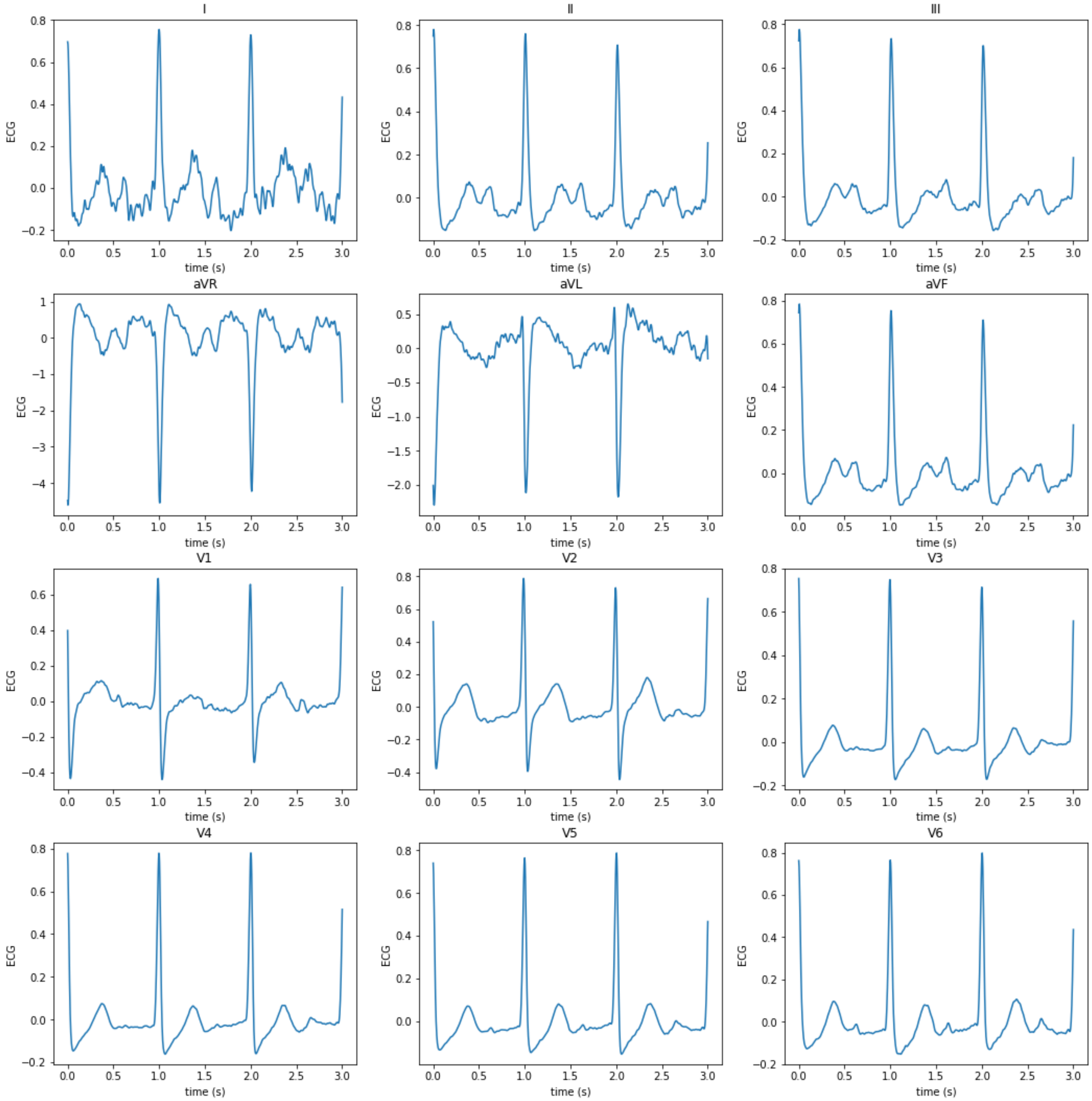

Supplementary Figure S 5. ECG waveform of I-AVB patient

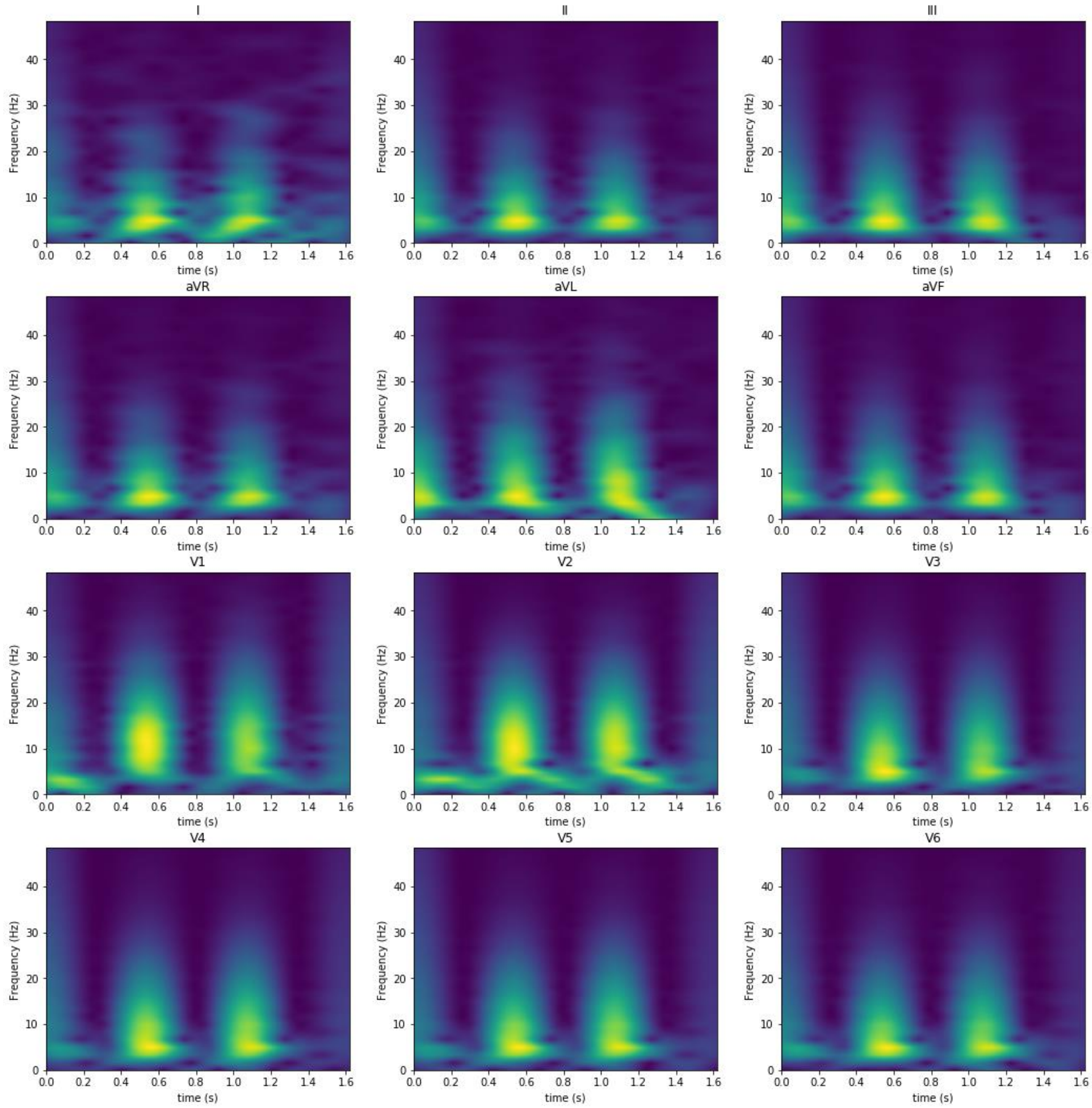

Supplementary Figure S 6. Time-frequency map of I-AVB patient

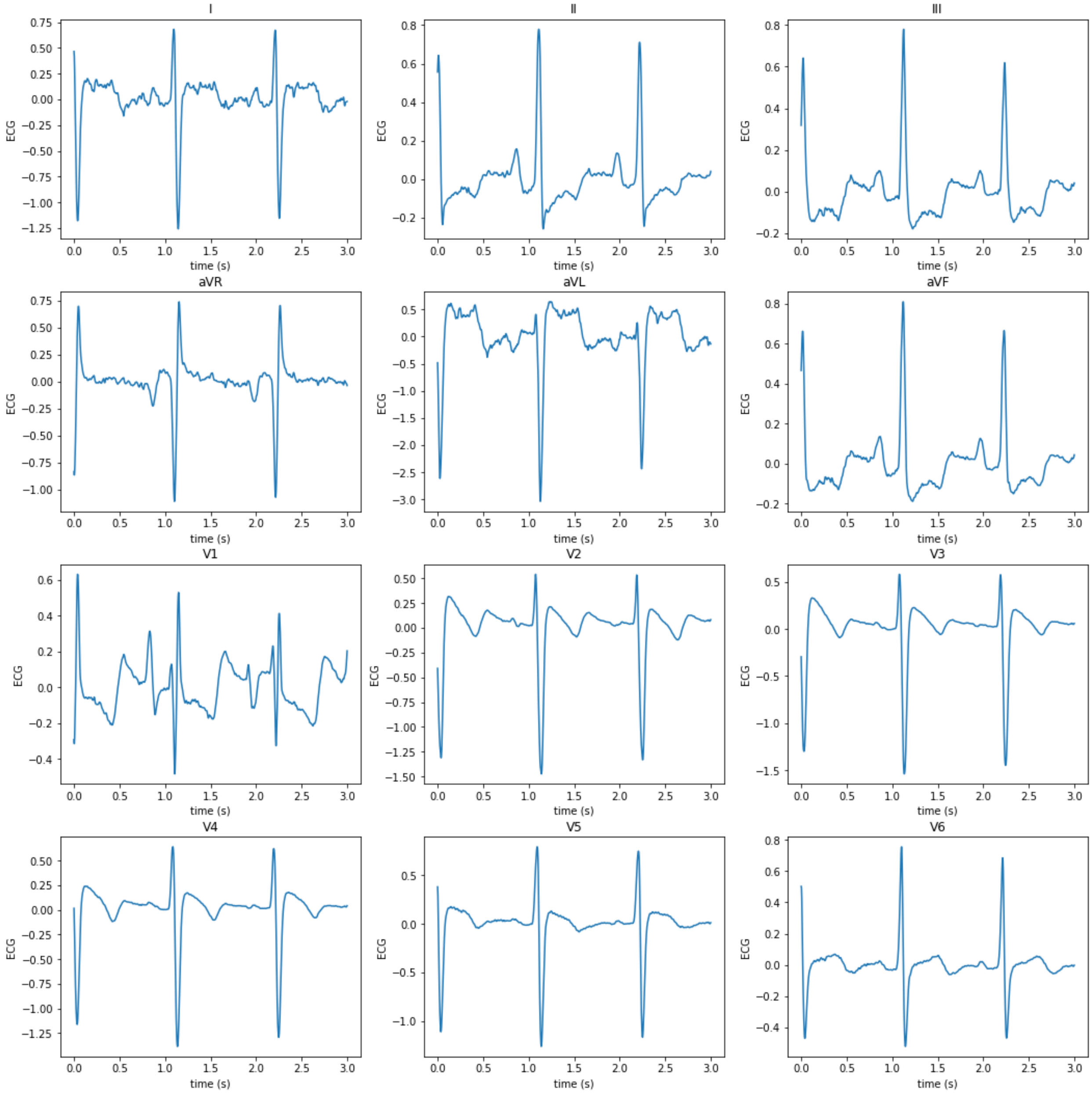

Supplementary Figure S 7. ECG waveform of PAC patient

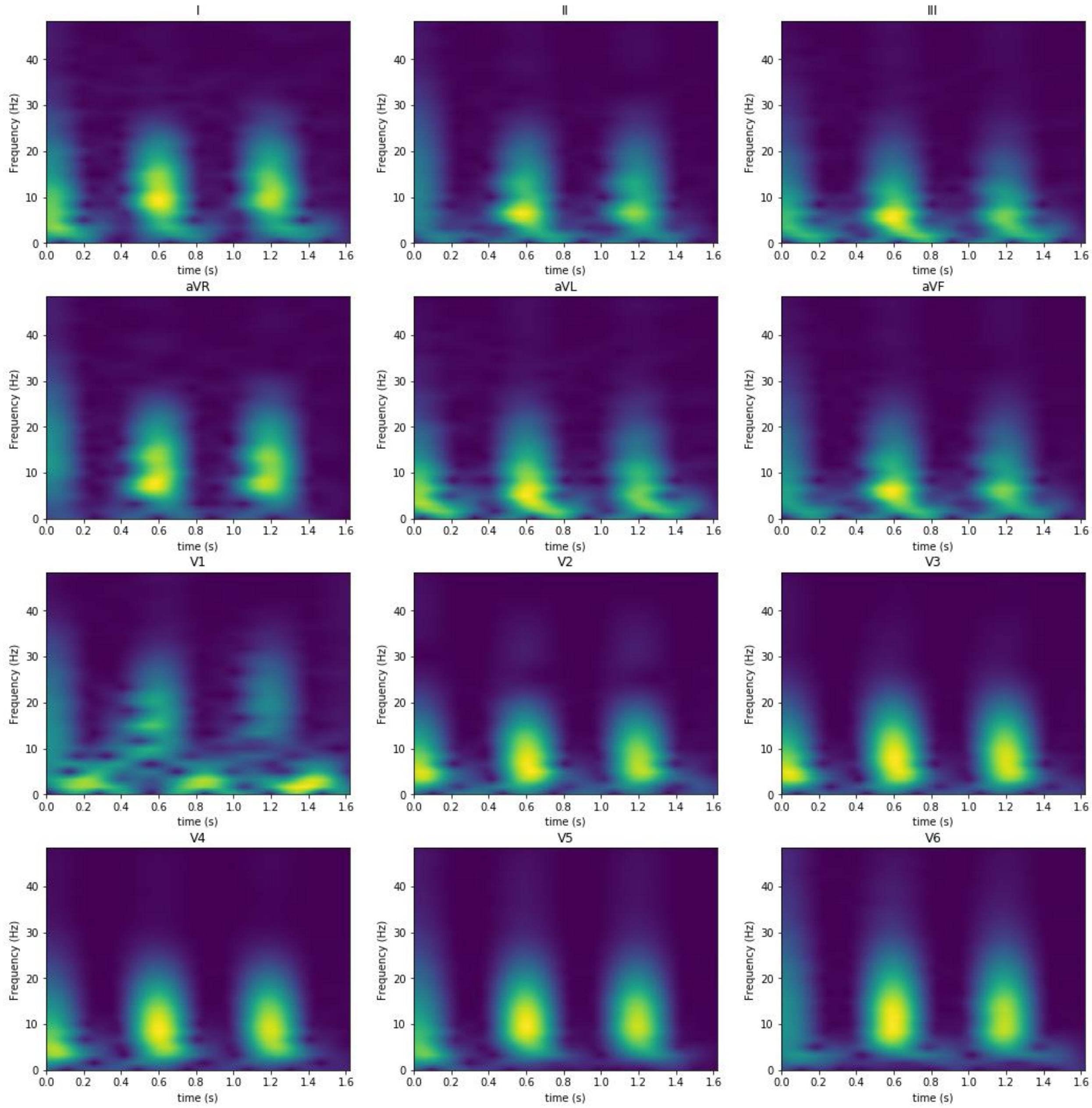

Supplementary Figure S 8. Time-frequency maps of PAC patient

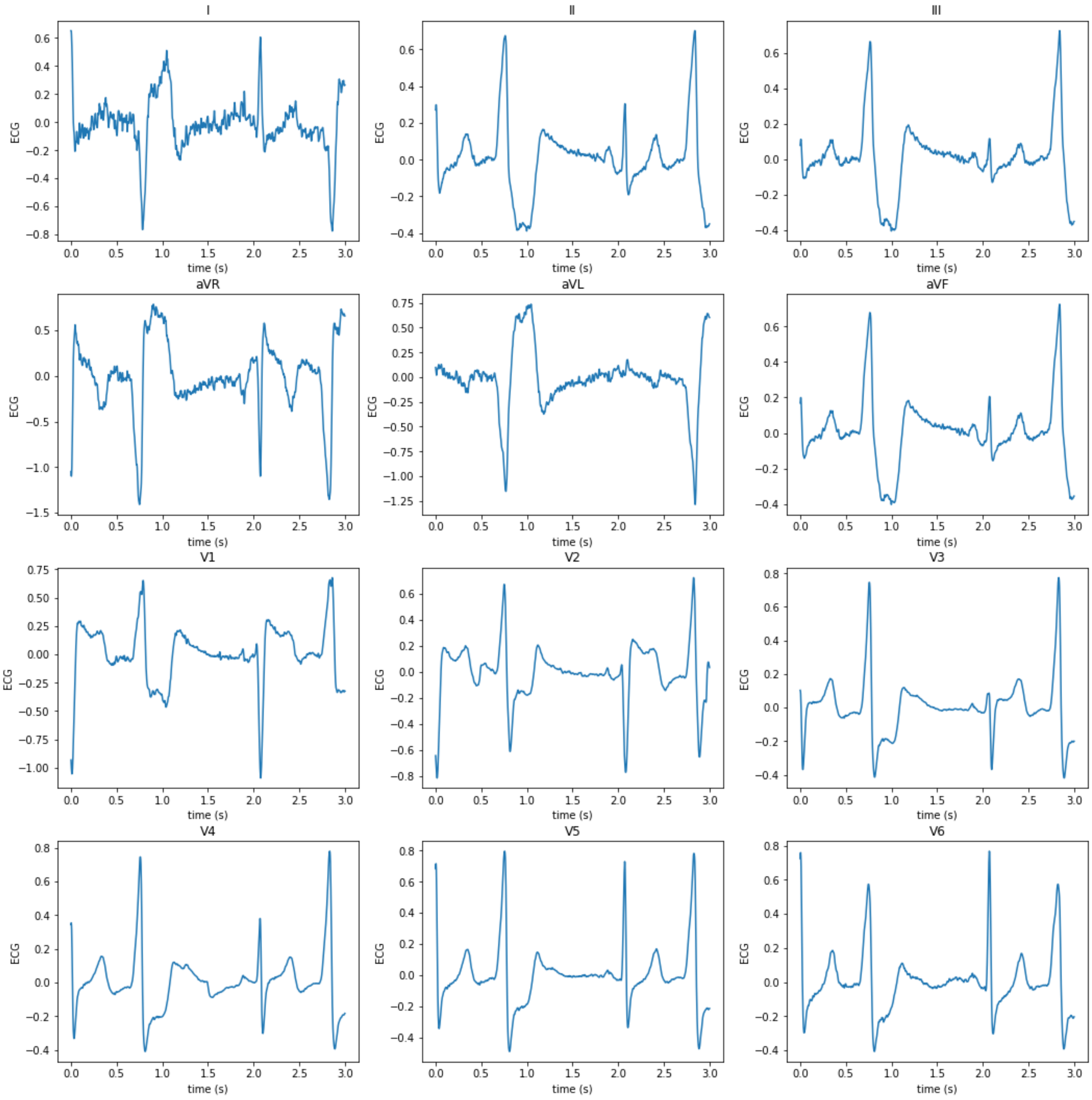

Supplementary Figure S 9. ECG waveform of PVC patient

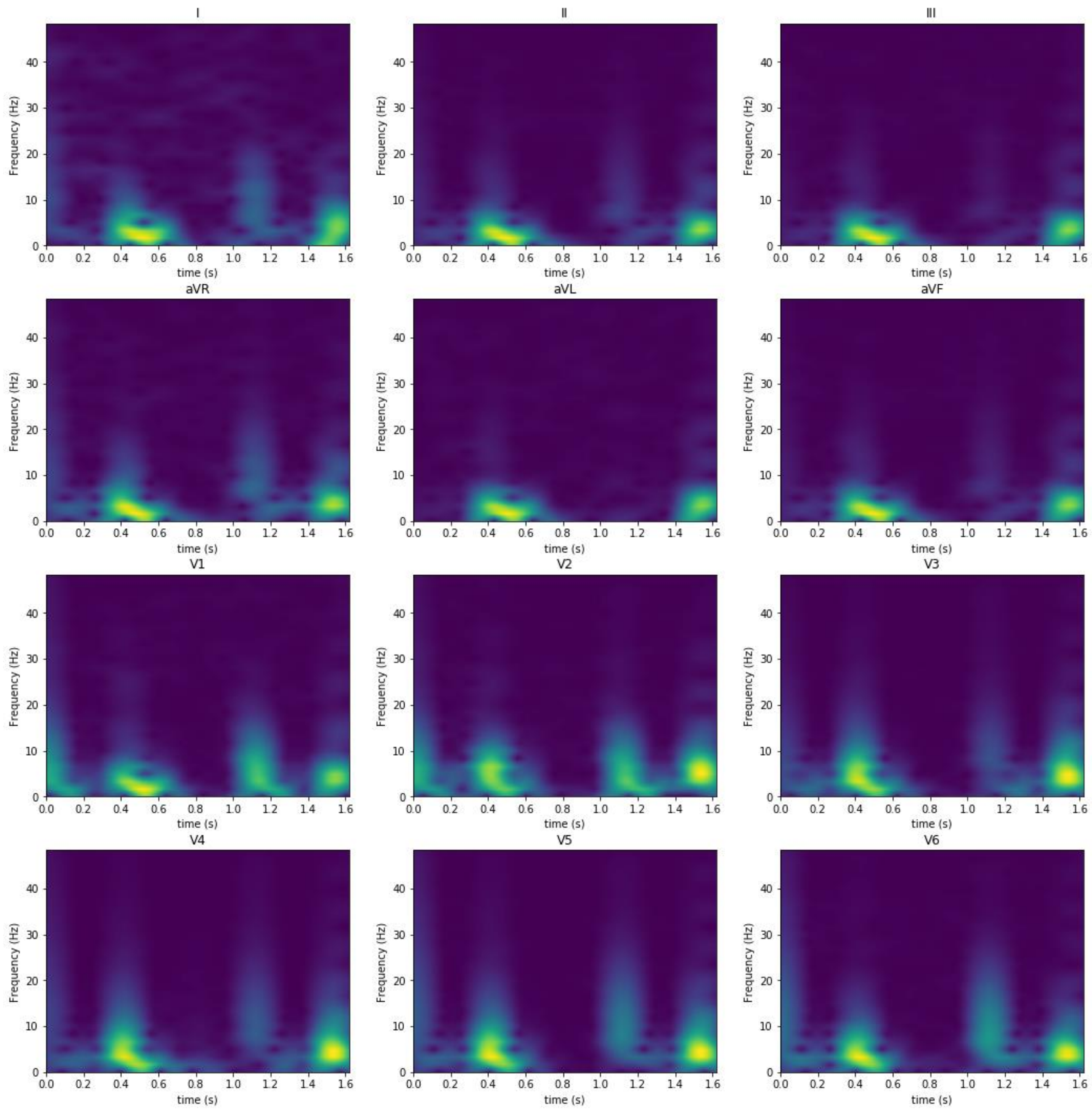

Supplementary Figure S 10. Time-frequency maps of PVC patient

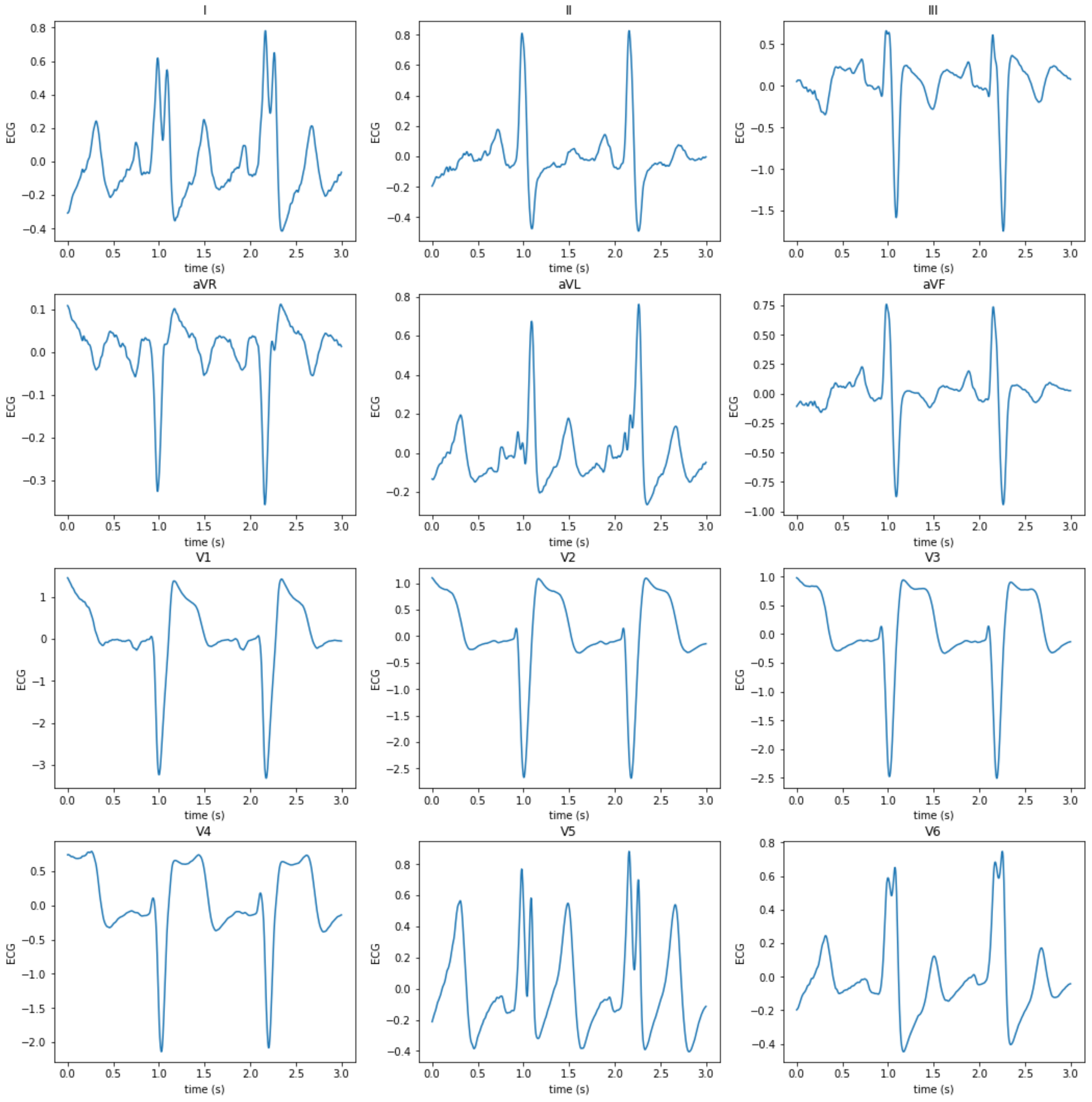

Supplementary Figure S 11. ECG waveform of LBBB patient

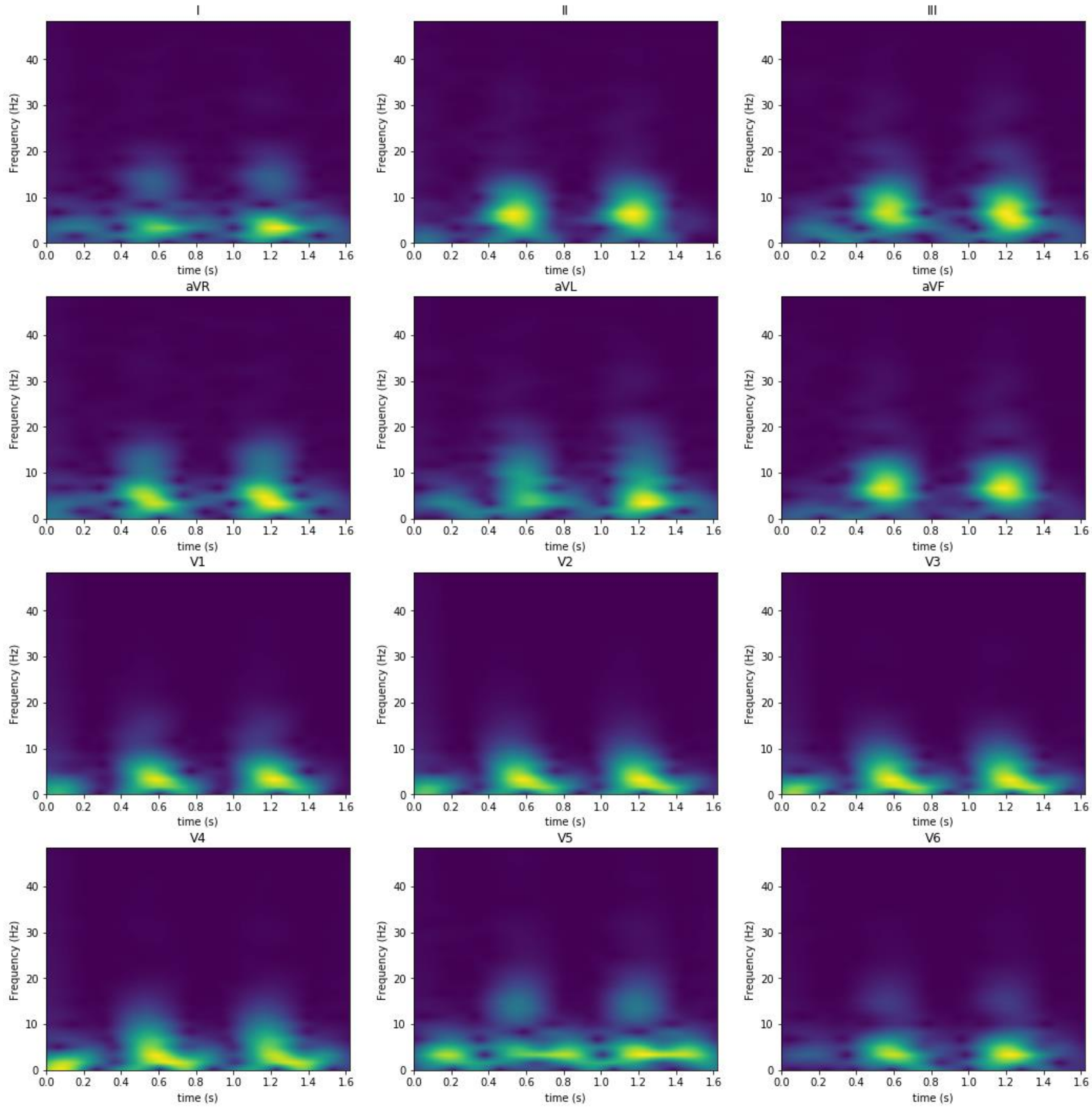

Supplementary Figure S 12. Time-frequency maps of LBBB patient

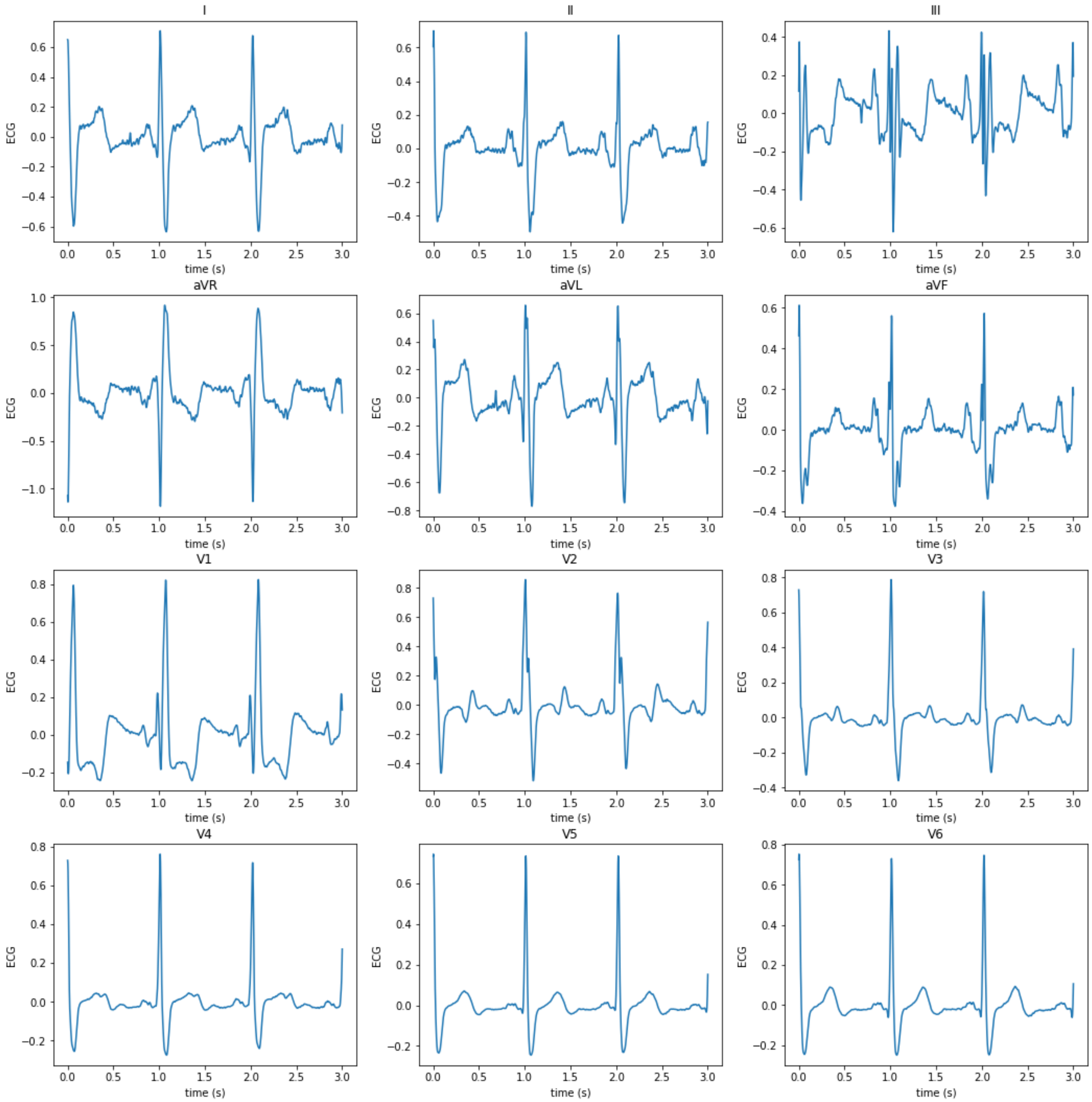

Supplementary Figure S 13. ECG waveform of RBBB patient

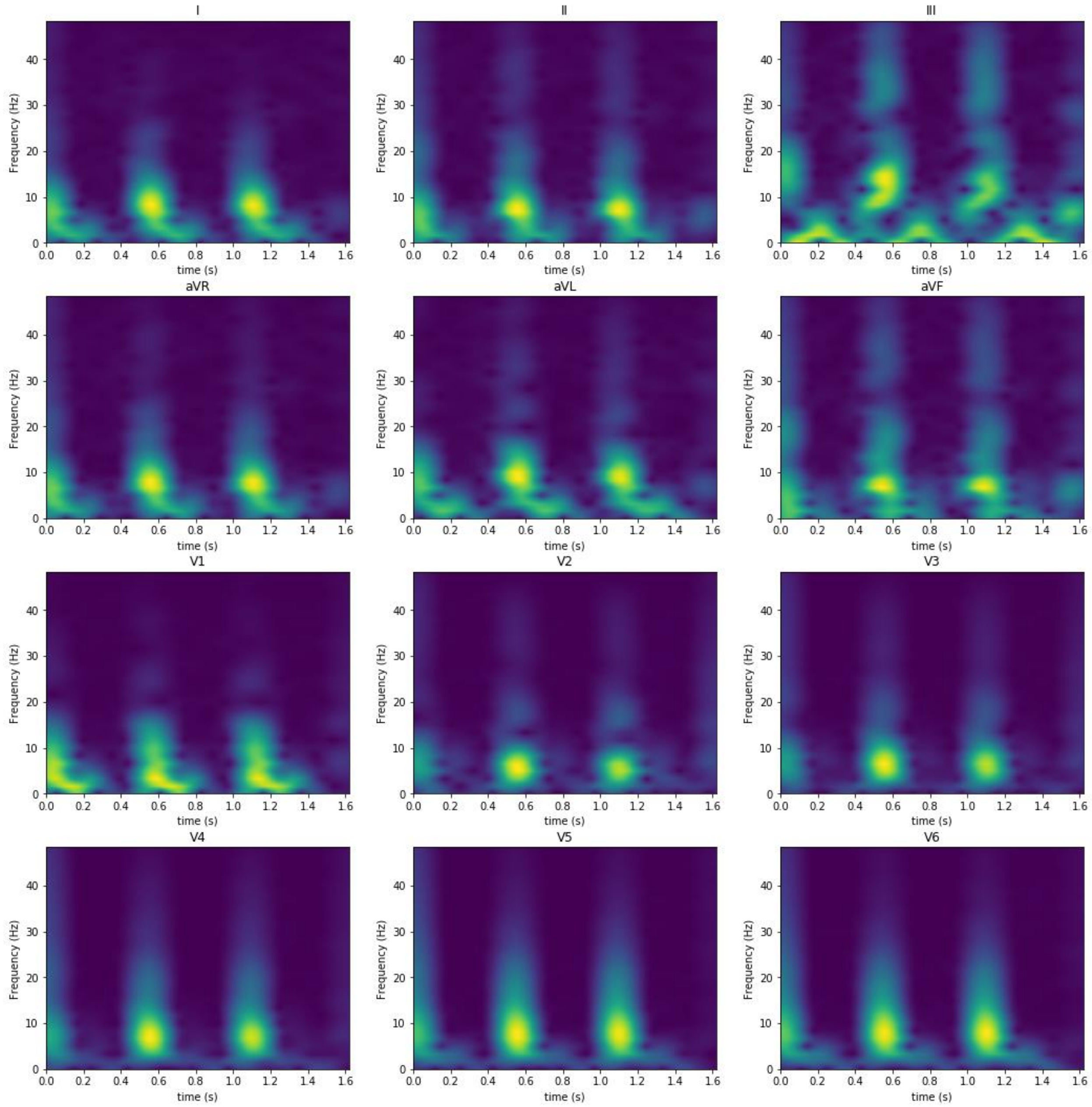

Supplementary Figure S 14. Time-frequency maps of RBBB patient

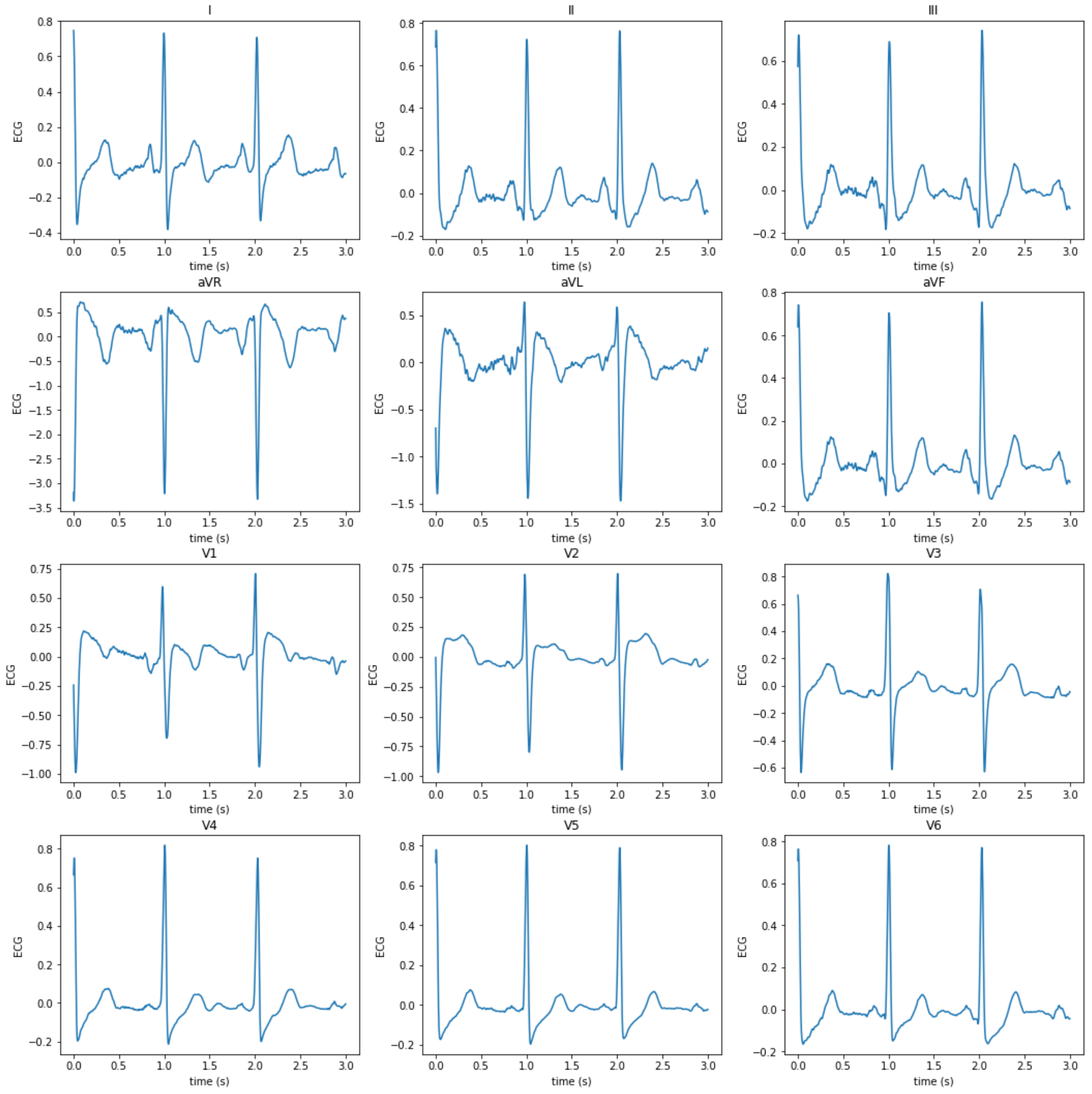

Supplementary Figure S 15. ECG waveform of STD patient

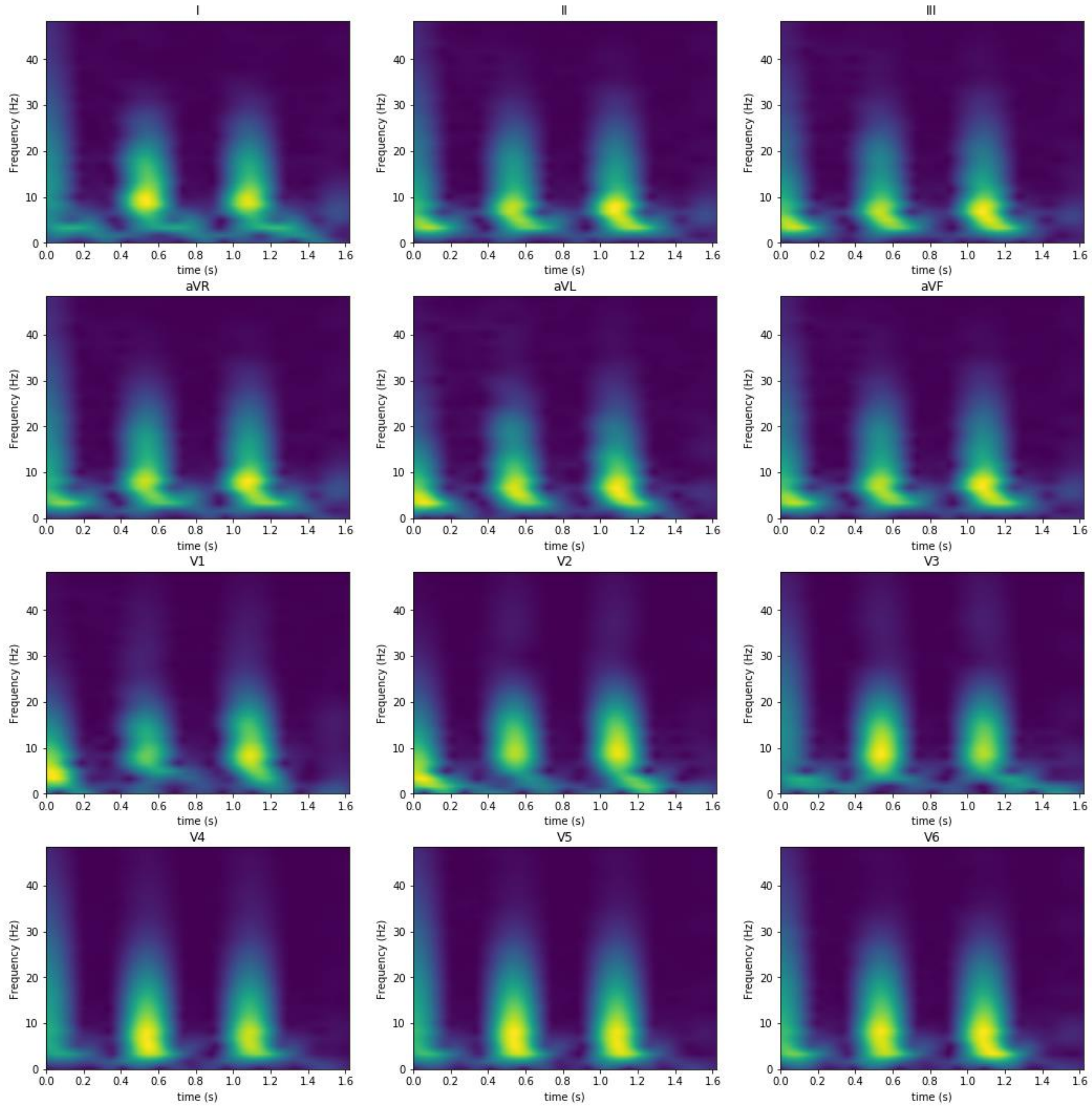

Supplementary Figure S 16. Time-frequency maps of STD patient

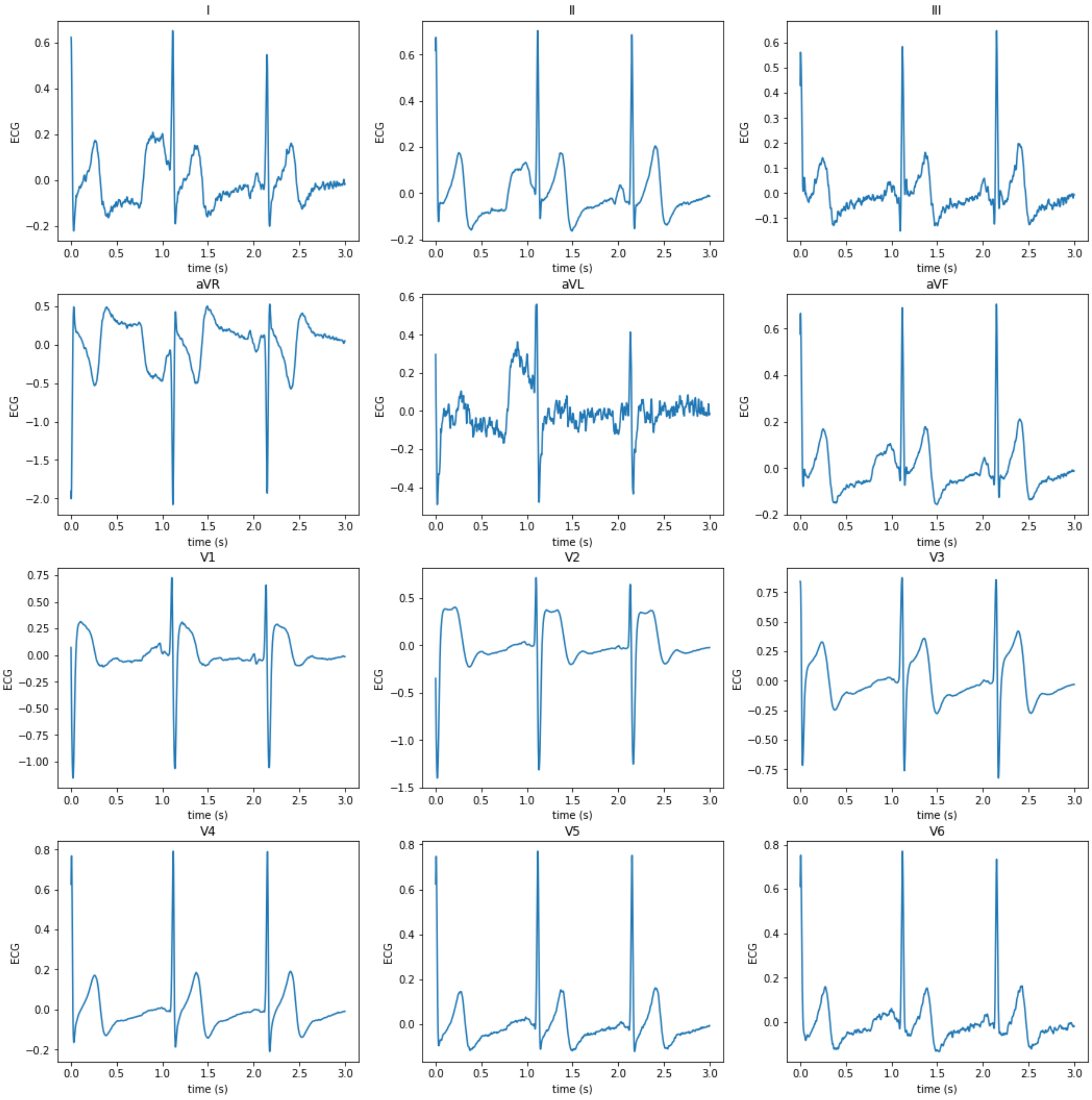

Supplementary Figure S 17. ECG waveform of STE patient

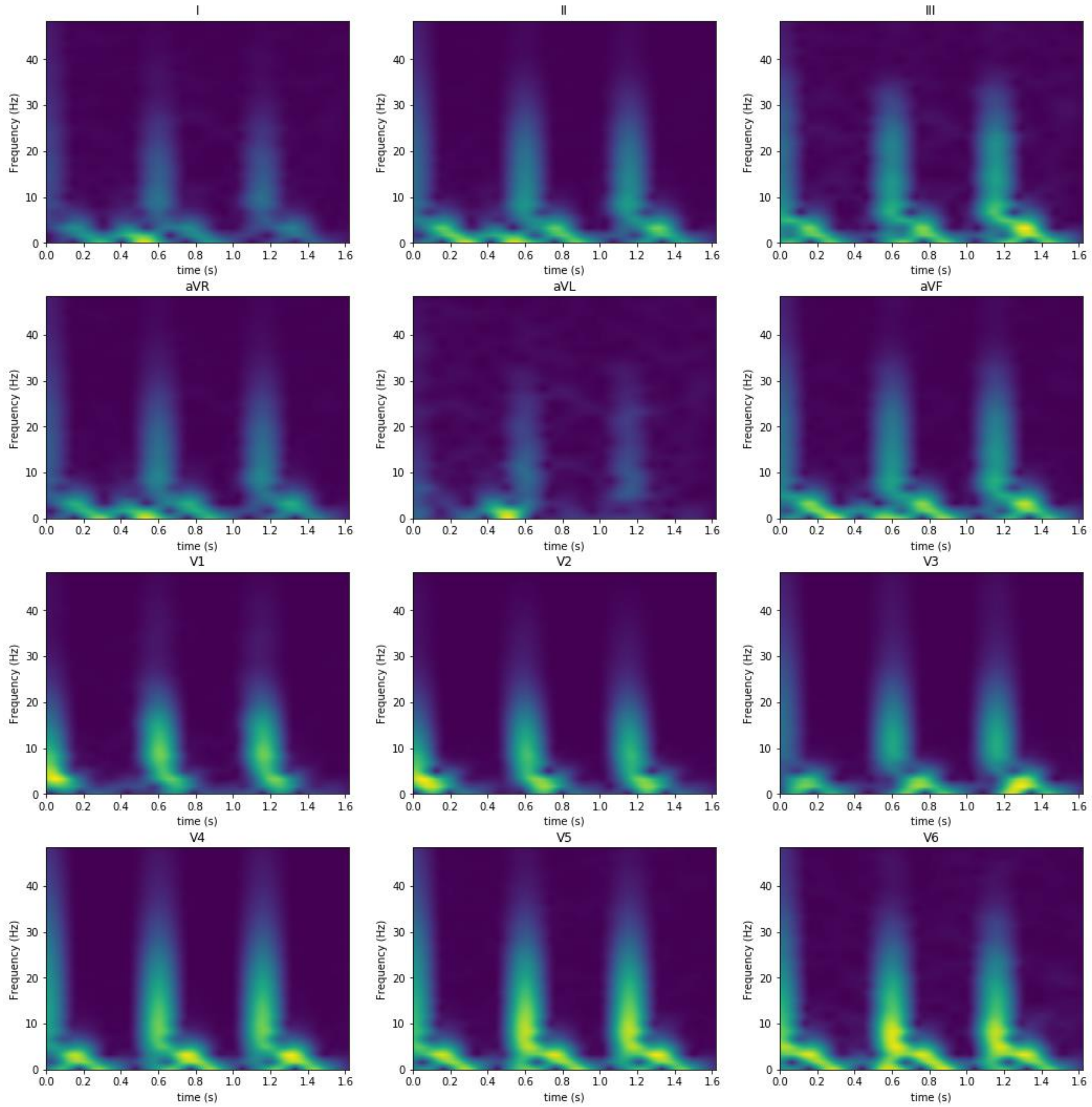

Supplementary Figure S 18. Time-frequency maps of STE patient
